# Evaluating the performance of ancient DNA genetic relatedness estimation methods using high-fidelity pedigree simulations

**DOI:** 10.1186/s13059-026-04016-y

**Published:** 2026-03-09

**Authors:** Maël Lefeuvre, Marie-Claude Marsolier, Céline Bon

**Affiliations:** 1 UMR 7206 - Eco-anthropologie (EA), Muséum national d’Histoire naturelle, CNRS, Université Paris Cité, Musée de l’Homme 17 place du Trocadéro, 75016 Paris, France; 2 UMR 9198 - Institut de Biologie Intégrative de la Cellule (I2BC), CNRS, CEA, Université Paris-Saclay, 1 Avenue de la Terrasse, 91190 Gif-sur-Yvette, France

**Keywords:** Genetic relatedness, Paleogenomics, Ancient DNA, Benchmarking, Pedigree, *In silico* simulations

## Abstract

**Background:**

Recent advancements in paleogenetics, coupled with the emergence of dedicated statistical methods have, in recent years, streamlined the detection of close genetic ties from ancient DNA samples, leading to a substantial surge in scientific publications emphasising the reconstruction of genealogies within archaeological funerary contexts. However, while these methods all claim aptitude for addressing the inherent biases of ancient DNA, assessing their practical reliability can often be challenging, particularly in case studies involving few and/or poorly preserved samples. Furthermore, the genetic heritage and cultural practices of the population studied (e.g., inbreeding, endogamy) are factors which are often both complex to estimate and capable of impacting the accuracy of these methods.

**Results:**

We present an in-depth comparative study of six ancient DNA genetic relatedness estimation methods to precisely delineate their respective performance and behaviour across a range of five biological parameters: sample coverage, use of *post-mortem* damage correction methods, human contamination, genetic diversity, and inbreeding. To this end, we develop BADGER (Benchmark Ancient DNA GEnetic Relatedness), an automated pipeline and software which first simulates pedigrees using randomly selected present-day individuals from the 1000-genomes dataset, and subsequently generates raw ancient DNA sequence data for each individual within these trees.

**Conclusions:**

The results of this benchmark enable us to discuss the individual strengths and limitations of these methods, propose a set of prescriptions to consider when interpreting their results and demonstrate that their reliability cannot be predicted from sample coverage alone, and may be subject to multiple sources of bias.

**Supplementary Information:**

The online version contains supplementary material available at 10.1186/s13059-026-04016-y.

## Background

The study of DNA-based kinship relationships within archaeological contexts has been the subject of sustained interest, in line with the improved cost-effectiveness of ancient DNA sequencing techniques. As a result, many publications have chosen to place this type of analysis at the forefront, to highlight the funerary practices, lineal descent and/or residence rules, or kinship-based social inequalities found within past societies [1–9]. This partial shift, from solely studying large-scale population dynamics to an increased focus on deciphering finer-grained genetic relatedness between archaeological samples, has been bolstered by the continuous development of dedicated computational methods to estimate genetic relatedness from low-coverage ancient DNA shotgun sequencing data. While such methodological advances have followed closely on the heels of the *technical* advancements in genome-wide analysis of ancient DNA samples [10–18], research efforts have intensified in recent years, with at least eight novel or updated genetic relatedness estimation software published between 2022 and 2024 [19–26].

These methods all aim to provide estimates of relatedness through a direct or indirect calculation of a coefficient of relationship *r*, a summary statistic which encapsulates the probability that two alleles, each randomly sampled from a given pair of individuals, are identical by descent (IBD). Calculating this metric is generally a trivial task when applied to modern, high-coverage, whole-genome sequencing data (WGS). The field of paleogenomics, however, is riddled with specific challenges that are ultimately linked to DNA degradation over time, often resulting in data of low coverage, exhibiting characteristic patterns of *post-mortem* damage (PMD) and an increased sensitivity to human and environmental contamination [27]. Hence, to overcome the sparseness of ancient DNA data, all of the previously cited genetic relatedness estimation methods fall in either one of two heuristics to estimate *r*, namely, i) the use of randomly sampled pseudo-haploidised genotypes [13, 15, 16, 19–22, 24–26], or ii) the estimation of IBD probabilities from genotype likelihoods [10–12, 14, 17, 18, 23]. These two primary strategies evidently possess inherent strengths and weaknesses, the former being theoretically applicable to samples of very low coverage, while the latter is expected to enhance accuracy, albeit at the expense of stricter requirements regarding sample sequencing depth, given its use of diploid information. Furthermore, these methods will often compensate the loss of statistical power introduced through these heuristics by either i) normalising the results across all available samples [20, 21, 25, 26], or ii) the use of a genotype reference, through the introduction of reference samples or allele frequencies [10–15, 17, 19, 22–24]. Note that these two latter subcategories of methods will be hereupon defined as “cohort-normalisation” and “reference-based” methods, respectively.

Regrettably, and in the midst of this relative effervescence, these methods, both the well-established and the newly developed were, for the most part, largely adopted despite any prior in-depth examination of their respective accuracy, bias and best practices required for their use. Indeed, to our knowledge, and beyond the customary tool-specific benchmarks that appear in the respective publications of these methods, efforts to provide the field with systematic benchmarks are sparse, and have only been carried out in recent years [28, 29]. These first two publications already provide valuable general estimates of the average sequencing depth required to apply these methods, and offer a clear comparison of the respective strengths and weaknesses of pseudo-haploidisation methods *versus* those employing genotype likelihoods. Aktürk et al. [29] additionally offers vivid glimpses into the impact of inbreeding, and the effect of supplying these methods with inaccurate reference allele frequencies, which are two challenging sources of bias in paleogenomic studies. Yet, while these previous endeavours have provided the field with invaluable insights, their contribution, as a whole, remains insufficient to fully delineate the applicability of these methods in more realistic use-cases and archaeological contexts, owing to several critical shortcomings in their design, which include – but are not limited to – the following points:

- First and foremost, both Marsh et al. [28] and Aktürk et al. [29] extensively rely on downsampling analysis-ready BAM files to generate test datasets for their benchmark. While this approach carries the benefit of being computationally effective, it also fails to capture any bias which may be introduced from aligning and pre-processing data that is lowly covered, highly damaged, and/or contaminated – as is typically the case in ancient DNA studies.
- Additionally, Marsh et al. [28] applied their benchmark on real ancient DNA datasets. This design choice effectively negates any risk of introducing bias through simulated data, but forces the authors to merely make weak assumptions regarding what should or should not be considered a true positive result, considering the fact that no ground truth can be established from these datasets.
- Finally, Aktürk et al. [29] address the latter point accordingly, through the use of simulated data. However, their designed protocol ultimately compels them to provide several of their tested methods with a combined dataset, containing between 2468 and 4458 unrelated pairs. Ultimately, this protocol effectively simulates a use-case where tens to a hundred of exploitable samples are available, which is seldom the case in archaeological contexts. We suspect that this key design choice may yield overly confident results, given that sample size is often a critical parameter in statistical analyses and methods.

Hence, to address these limitations, and complement the current state of knowledge in estimating genetic relatedness within archaeological contexts, we present the results of an additional benchmark, focusing on evaluating these methods in the context of a more realistic and more frequently encountered scenario, i.e. by simulating a small, densely connected family tree, containing 31 pairs of unrelated individuals out of a total of 55 possible pairwise comparisons ($n=11$ individuals). We evaluated the impact of sequencing depth, contamination, population diversity and close inbreeding on the performance of six previously published ancient DNA genetic relatedness estimation methods: “correctKin”, “GRUPS-rs”, “KIN”, “READ”, “READv2”, and “TKGWV2” (Table 1). (For the sake of transparency, note that the software “GRUPS-rs” was developed by the main authors of this paper – see Table 1 and Section Competing interests). Contrary to the previous endeavours of Marsh et al. [28] and Aktürk et al. [29], we additionally focus on performing independent simulation replicates of whole-genome shotgun sequencing data, at low to very low coverages (i.e. [0.02–1]X range), and without the use of data downsampling. Note here that we also focus solely on methods utilising pseudo-haploid data. This is partly a design choice that is linked to the higher requirements of these tools in terms of average runtime, but also due to the fact that the performance of genotype likelihoods methods such as lcMLkin and NgsRelate were both already extensively tested in both Marsh et al. [28] and Aktürk et al. [29], and were already demonstrated to behave poorly under the specific conditions we are emulating here. Namely, (i) methods such as lcMLkin or NgsRelate-v2 generally expect data that is locally of at least 2X coverage, using independant loci. Obtaining such data at sequencing depths far lower than 1X is achievable when using capture-data, or when benchmarking these tools from downsampled data, but is expectedly harder when using WGS data in the range [0.02 – 1]X. Besides, (ii) the previous works of Marsh et al. showed that the consistency of the software NGSRemix, NgsRelate and lcMLKin sharply declined when the downsampled coverages were lower than 1.03X, whereas our protocol intends to assess performances in the range [0.02 - 1]X [28]. Finally, (iii) methods such as NGSRemix may require direct calculation of population-specific allele frequencies and ancestry proportions from the sample, which are expected to be rather imprecise, when applied on a cohort of merely 11 strongly related individuals.

**Table 1:** Description of the methods benchmarked in Marsh et al. [28], Aktürk et al. [29], and this study

Method	Original publication	Input data type	Benchmarked in
lcMLkin	Lipatov et al. [10]	Genotype likelihoods	[28]
lcMLkin (v2.1)	Altınışk [30]	Genotype likelihoods	[29]
NGSremix	Nøhr et al. [31]	Genotype likelihoods	[28, 29]
NgsRelate	Korneliussen and Moltke [11]	Genotype likelihoods	[28]
NgsRelate-v2	Hanghøj et al. [17]	Genotype likelihoods	[29]
“Kennett method”	Kennett et al. [3]	pseudo-haploid	[28]
READ	Kuhn et al. [16]	pseudo-haploid	This study | [28, 29]
READv2	Alaçamlı et al. [25]	pseudo-haploid	This study
KIN	Popli et al. [21]	pseudo-haploid	This study | [29]
TKGWV2	Fernandes et al. [19]	pseudo-haploid	This study | [28]
correctKin	Nyerki et al. [22]	pseudo-haploid	This study
GRUPS-rs	Lefeuvre et al. [24]	pseudo-haploid	This study

Within the overarching category of pseudo-haploidisation methods, the choice remains between utilising methods based on reference data, or those employing cohort normalisation. Selecting the most appropriate method within one of these two subcategories is often of strong significance in the field, as many archaeological case studies will often involve a small number of individuals at hand and/or lack the necessary context to define a precise and accurate reference dataset. As such, the present panel of candidate methods was selected with the objective of exemplifying a range of approaches to manipulating and leveraging these strategies:

- READ and READv2 – decidedly two of the most widely used methods within the field – alleviate the need of using reference data by calculating the average pairwise mismatch rate (PMR) between all pairs of individuals, and merely rectify systemic deviations caused by the population heterozygosity rate by dividing these PMR estimates by their corresponding median [16, 25].
- KIN endeavours to identify and estimate genetic relatedness from IBD tracts using a Hidden Markov Model (HMM) [21]. This strategy is purportedly more robust to the presence of long runs of homozygosity (ROH) – attributable to either a small population size, or recent inbreeding within the sample – but equally requires prior knowledge of the expected proportion of pairwise differences found between two unrelated samples within the population. Here KIN reportedly draws direct inspiration from READ, and models this parameter as the median of all available pairwise comparisons [21].
- GRUPS-rs similarly calculates pairwise mismatch rates, but departs from the use of median normalisation, and rather employs pedigree simulations in thousands of replicates from a reference panel of present-day human phased genotypes [24] (typically, genotypes originating from the 1000g-phase3 dataset [32]). This strategy negates the need of a cohort of individuals, instead relying on comparing the observed pairwise mismatch rate against theoretical expectations, and makes GRUPS-rs applicable to a single pair of individuals – distinguishing it from methods such as READ, READv2, and KIN.
- Alternatively, TKGWV2 implements a unique adaptation of the Queller & Goodnight (QG) estimator [33] for pseudo-haploid data [19]. Crucially, this backend algorithm entails the use of allele frequencies and, by extension, hinges on possessing comprehensive knowledge of the population’s genetic composition and diversity (which is seldom the case in real-world instances). The devised strategy, however, also makes TKGWV2 applicable to a single pair of individuals, and theoretically provides additional sensitivity, given that sharing a rare allele is more strongly indicative of close relatedness. (i.e. given a pair of diallelic genotypes with an allele frequency *p*, the single-locus relatedness estimate provided by QG’s algorithm is given as $r = 1 - \frac{1}{2p} - \frac{1}{2(1-p)}$ in case of a pairwise mismatch, but reduces to $r=1$ when both alleles are identical [34]).
- Finally, the software correctKin leverages principal component analysis as a clustering method, to delineate kindred individuals from the main cohort, and accounts for low coverage using a simple linear regression model [22]. Importantly, however, this approach still relies on having a substantial sample size at hand, and on the assumption that most individuals are unrelated. Thus, for many use-cases, best results are expected to be obtained when additionally providing correctKin with a panel of unrelated samples from a public dataset, as recommended by the authors [22].

Given these descriptions, we hereupon consider and define READ, READv2 and KIN as “cohort-normalisation” methods, whereas GRUPS-rs, correctKin and TKGWV2 are here considered as “reference-based” methods.

The benchmark developed for this research is here provided as an automated, self-contained software termed “BADGER” (**B**enchmark **A**ncient **D**NA **GE**netic **R**elatedness). This effort both ensures the reproducibility of the results presented here, but can also be repurposed by researchers, to evaluate the *expected* results of these genetic relatedness estimation methods, given the specific parameters of their dataset.

## Results

### Benchmark design and implementation

#### An overview of BADGER, an automated benchmark of ancient DNA genetic relatedness estimation methods using high-fidelity pedigree simulations

**Fig. 1:**
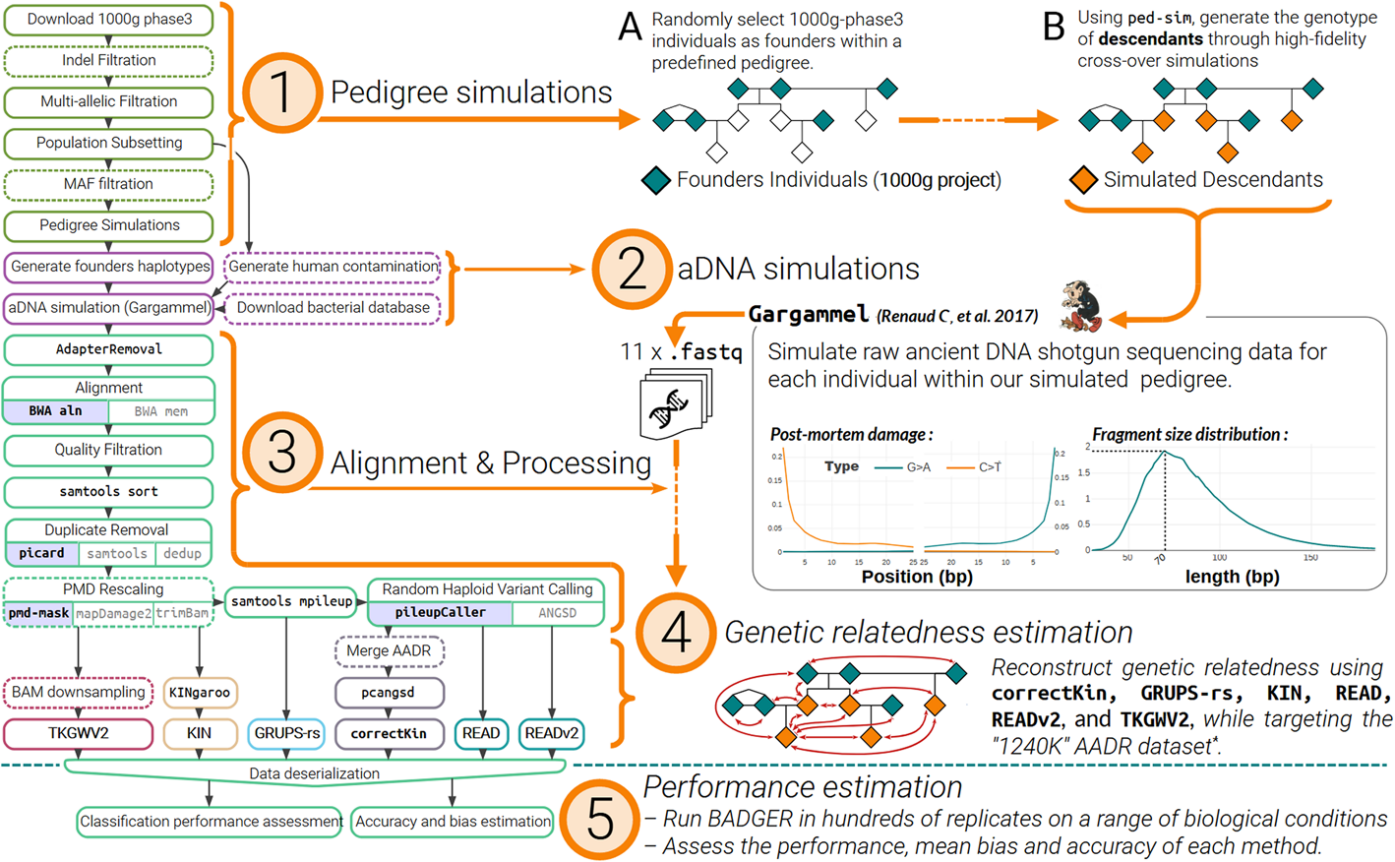
Simplified directed acyclic graph, and summary of the main steps composing the BADGER pipeline. Steps of the workflow where multiple alternative software or utilities are available for use are detailed accordingly, with the default selected software highlighted in pastel blue. (* *"1240K"* AADR dataset: [60].)

BADGER is an automated, user-configurable and scalable pipeline written in snakemake [35] and is designed to evaluate and compare the classification performance of multiple ancient DNA genetic relatedness estimation methods. Briefly, the overall workflow and architecture of BADGER, described extensively in Section Methods – “Description of BADGER’s simulation pipeline”, can be partitioned and summarised into five successive steps (Fig. 1):

1. *Pedigree simulation*: To generate its test data, BADGER first employs high-definition pedigree simulations, using the software Ped-sim [36]. Starting from randomly sampled individuals from the 1000g-phase3 project as a source of founder individuals, the genotypes of descendants are thus generated within the constraints of a predefined template family tree (Fig. 1(1)). Note that the founder individuals used during the pedigree simulations are always selected from a predefined (super)population label of the 1000g-phase3 dataset. Unless stated otherwise, all simulations presented in this benchmark were applied using the CEU population (*Utah residents with Northern and Western European ancestry*) as the source for founder individuals.
2. *aDNA sequence data simulation*: Using the software gargammel [37], BADGER then converts the genotype data of every pedigree member into raw ancient DNA sequence data. Here, the characteristic patterns of *post-mortem* damage and low sequencing depth, typically exhibited by ancient DNA samples, are simulated according to user-defined parameters (Fig. 1(2)). Notably, BADGER can simulate a specified proportion of modern human contamination at this step, through the incorporation of exogenous sequences, sampled from a randomly selected subset of 1000g-phase3 individuals.
3. *Alignment and pre-processing*: Following best practices [38, 39], the raw sequence data of every simulated individual goes through the established process of sequence alignment, quality control, and variant calling required to obtain analysis-ready files (Fig. 1(3)). Though computationally more intensive, this design choice allows BADGER to fully capture any bias potentially introduced by these standard pre-processing steps.
4. *Genetic relatedness estimation*: Finally, BADGER provides every tested method with the generated analysis-ready files, along with any required input, in an attempt to infer close genetic ties between these simulated samples (Fig. 1(4)).
5. *Performance estimation*: Following the application of steps 1–4 in multiple replicates, BADGER evaluates the performance and accuracy of each method by comparing their respective predictions with the genetic ties initially defined by the template pedigree provided to Ped-sim (Fig. 1(5)). Particularly, the general performance of every method is summarised using the Uniform Ordinal Classification Index (UOC) [40], a metric which notably takes into account the constitutive order separating every degree of relationship, and is bound between 0 and 1, with 0 implying optimal classification performance (see Section Estimation of classification performance). Accuracy and bias are also calculated using normalised estimates of the Root Mean Square Deviation (nRMSD) and Mean Bias Error (nMBE) of the raw relatedness coefficient (*r*-coefficient) estimates produced from each method (see Section Average accuracy and bias of relatedness coefficients, Additional file 1: Table S1).

#### Benchmark parameter space

**Fig. 2:**
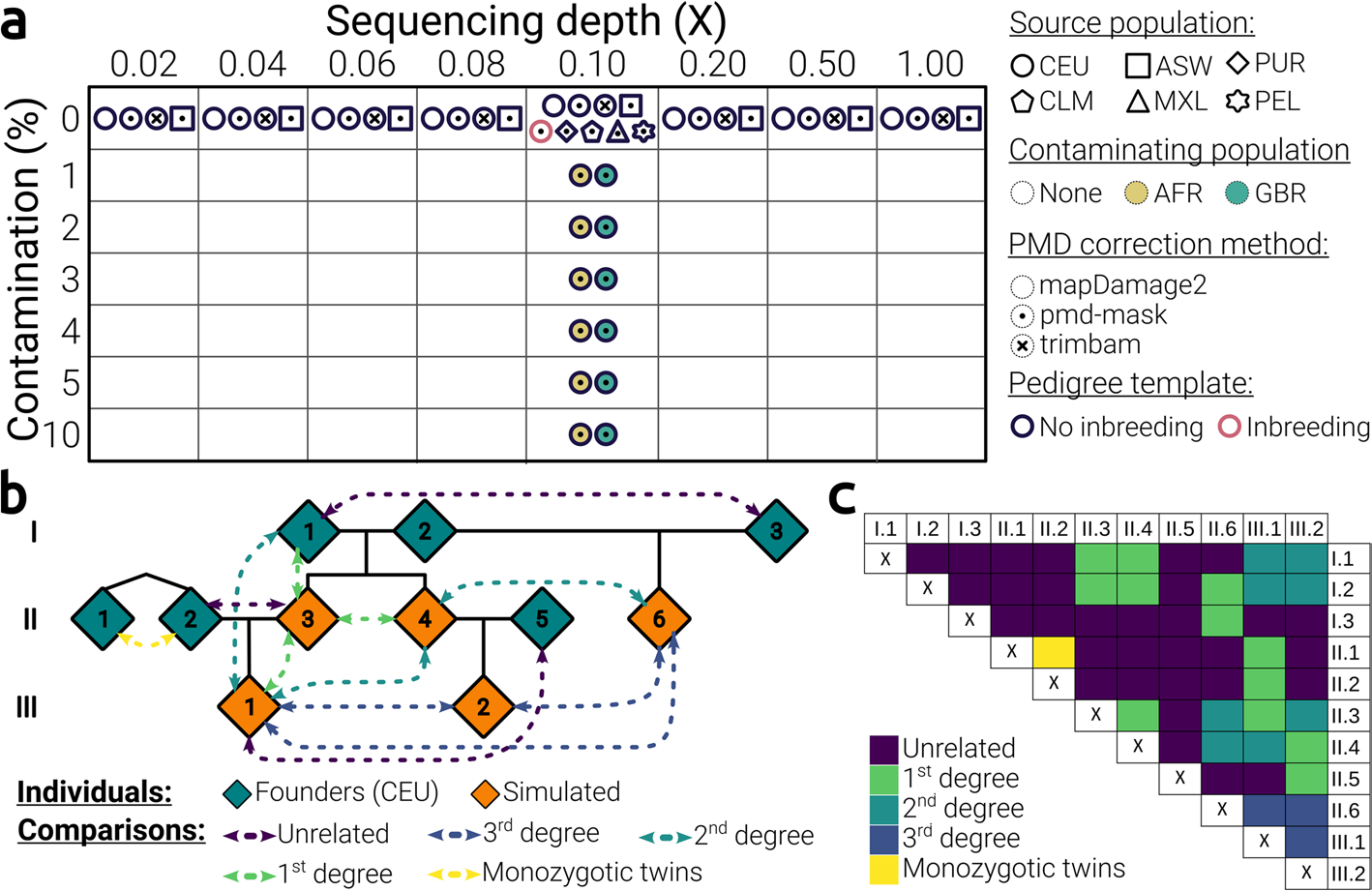
Parameter space and key design choices of the presented benchmark. **a** Summary of the range of parameters evaluated in this study. **b** Diagram of the main simulated pedigree featuring the pairwise relationships investigated during the benchmark. **c** Pairwise matrix of the relationships defined by the simulated pedigree shown in (**b**). All pairwise relationships contained within the input pedigrees used throughout this study are thoroughly described in Additional file 1: Table S2

We here assess the performance of READ, READv2, TKGWV2, KIN, correctKin, and GRUPS-rs, against a broad spectrum of biological parameters, using BADGER. This evaluation was achieved by implementing *n*=100 simulation replicates for each of the described scenarii (Fig. 2A). Unless specified otherwise, all simulations were performed using the same input pedigree, investigated an identical subset of pairwise relationships (Fig. 2B and C, Additional file 1: Table S2), and selected the CEU population as the source of founder individuals for the pedigree replicates. We investigated the impact of the following five biological parameters on the performance of each method:

##### Sample coverage

The impact of sample coverage was first investigated by applying BADGER across eight values of average sequencing depth, ranging from 0.02 to 1X, using CEU individuals as a source population.

##### PMD-correction methods

In order to mitigate confounding effects from *post-mortem* damage, direct modification of aligned sequences is often implemented when pre-processing ancient DNA samples. In such cases, a popular strategy resides in directly *masking* both extremities of reads or, at a finer level, selectively masking potential sites of DNA damage [41, 42]. This type of masking approach is here implemented as a default preprocessing step in BADGER, and used throughout this benchmark, through the use of the program pmd-mask, an in-house software designed to selectively mask potential $C>T$ and $G>A$ misincorporation sites at the 5’ and 3’ ends of reads, respectively (Additional file 2: Section 2 – “Description of the pmd-mask command line utility”). Similarly, the trimBam module of the bamUtil tool suite [41] applies exhaustive masking to both ends of the reads, up to a user-defined length threshold (typically 10bp, for non UDG-treated data). This second approach is more conservative than pmd-mask in that it masks the entire extremities, without distinguishing nucleotides that are locally more susceptible to deamination, from those that are less so. However, the process of PMD-correction is also quite commonly addressed through the use of *PMD-rescaling* approaches, i.e. methods which recalibrate base quality scores according to the probability that the genotype is derived from PMD-driven misincorporations [43–45]. Here, as these latter methods were recently demonstrated to exacerbate reference bias [42], we sought to directly compare the impact of these two alternative PMD-correction strategies when attempting to assess genetic relatedness. Thus, we applied BADGER across the same range of [0.02–1]X sequencing depths, this time incorporating either the PMD-rescaling software “mapDamage2” [43], or the trimBam software [41] (taking a threshold of 10pb) as alternative methods of PMD-correction during BADGER’s pre-processing stage (Fig. 1(3)), and compared the performance of each method under these conditions to those previously obtained through the default use of pmd-mask.

##### Contamination

We then studied the impact of six rates of modern human contamination, ranging from 1 to 10%, at a set sequencing depth of 0.1X, and using CEU individuals as a source population. Note that the scenario considered here involves contamination stemming from a *single* contaminating individual, with all pedigree samples exhibiting equal levels of contamination. Likewise, methods featuring contamination correction (i.e. GRUPS-rs, KIN [21, 24]) were not parametrised accordingly, in an effort to assess the impact of undetected or inaccurately estimated contamination. In addition, we evaluated the effect of the genetic proximity between the individuals comprising the pedigree, and the contaminating individual, by alternatively selecting as a source of contamination either individuals belonging to the African super-population (AFR), or individuals from the GBR population (*British in England and Scotland*), which is comparatively genetically closer to the source population used within the pedigree population (CEU).

##### Genetic diversity and reference panel bias

We applied BADGER at a constant sequencing depth of 0.1X, this time employing either one of five admixed American populations, with varying proportions of European (EUR) ancestry, as an alternative source of founder individuals (namely: *PUR*: *Puerto Rican in Puerto Rico*, *CLM*: *Colombian in Medellin, Colombia*, *MXL*: *Mexican Ancestry in Los Angeles, California*, *PEL*: *Peruvian in Lima, Peru*, *ASW*: *African Ancestry in Southwest United States*). For the ASW population, these simulations were additionally applied across the entire defined range of sequencing depths (i.e. [0.02 – 1]X), in order to assess the extent to which this source of bias interacted with sample coverage. Note here that all methods utilising allele frequencies or reference individuals (i.e. GRUPS-rs, correctKin and TKGWV2) were provided with the same EUR reference data, as in the other conditions. Thus, for READv2 and KIN, we straightforwardly evaluate the impact of applying these methods on admixed individuals, as they do not require the use of reference data. For correctKin, GRUPS-rs and TKGWV2 however, we concurrently evaluate the impact of incorrectly representing the genetic diversity of the population under study, as the reference data provided to these methods remains constant throughout all simulations.

##### Inbreeding

The impact of providing each method with closely inbred individuals was assessed by applying BADGER with an alternative pedigree, containing three additional inbred individuals, originating from the mating of either two full-siblings, two half-siblings or two first-cousins (Additional file 2: Fig. S1). Here, two types of analysis are performed, with the objective of evaluating two distinctive sources of bias, which may arise when applying these methods to inbred individuals:

1. To examine the way each method predicts comparisons involving an inbred individual, we analysed a first condition, where all individuals composing the pedigree are given as input, and nine comparisons involving one of the three inbred individuals are evaluated (Additional file 2: Fig. S1). To suppress any effect caused on the normalisation procedure of KIN and READv2 by the addition of three individuals, these two methods were in this case executed using a fixed normalisation value. This set value was calculated by taking the median of the medians obtained from all the previously generated outbred 0.1X simulation replicates (e.g.: –-norm-method ’value’ –-norm-value 0.2679719).
2. For correctKin, KIN and READv2 specifically, we then evaluate the impact of adding a *single* inbred individual to the cohort of tested individuals on their internal normalisation procedure, considering that these methods utilise the entire provided cohort of samples to calculate pairwise genetic relationships. To this effect, we consecutively test three alternative pedigree topologies, where only one out of the three inbred individuals (III.3, III.4 and IV.1) is included in the tested cohort (Additional file 2: Fig. S2). The classification performance of 13 outbred comparisons, identical to those assessed in the previous analyses (Fig. 2B), is then measured.

The results of these tests are here summarised, presented and analysed mainly using the UOC values calculated by BADGER. Sensitivity, specificity, balanced accuracy and F1-scores across all parameter configurations, methods and expected relationships were also calculated from the raw confusion matrices, using the caret R package [46], and are exhaustively reported in (Additional file 1: Table S3).

### Benchmark results

#### Impact of average sequencing depth

**Fig. 3:**
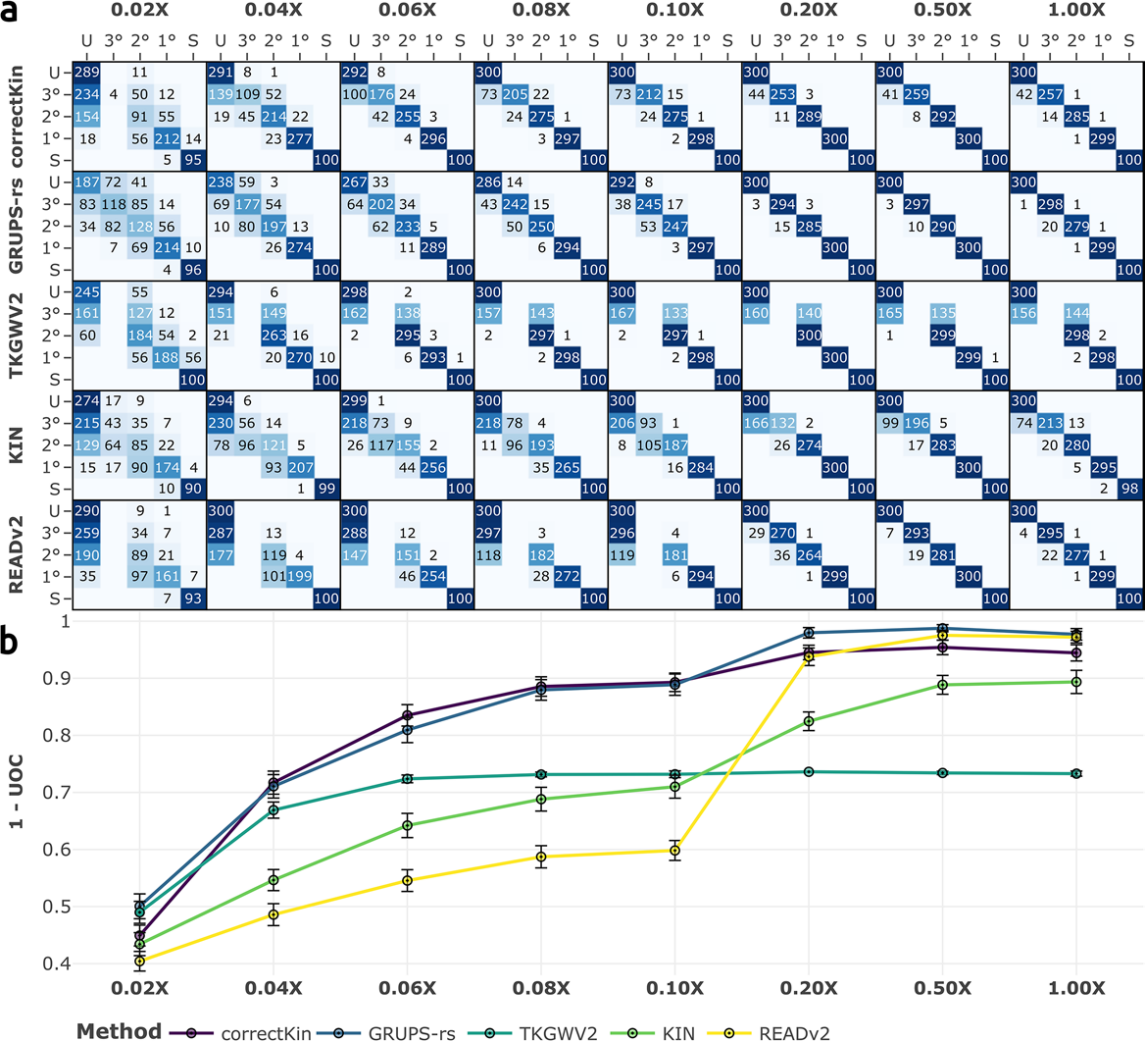
Benchmark results across increasing values of sequencing depth. **a** Confusion matrices of the five methods examined, confronting the expected and predicted relationships described in Fig. 2b. Expected and predicted values are displayed in rows and columns, respectively. “1°”, “2°”, “3°” correspond to first-, second-, and third-degree relationships, respectively, “U” corresponds to “unrelated individuals”, and “S” to “self” (monozygotic twins). **b** UOC values, summarising the classification performance of each method for the considered sequencing depths. Higher values of $1-UOC$ indicate higher performance. Error bars represent 95% confidence intervals around the mean UOC values, calculated from the standard deviation, and using normal approximation

We first studied the performance of correctKin, GRUPS-rs, TKGWV2, KIN and READ, and READv2 across eight average sequencing depths, ranging from 0.02X to 1X, using the default software pmd-mask for PMD correction (Fig. 3; Additional file 2: Fig. S3). Here, GRUPS-rs and correctKin displayed the highest average performance across all coverages, with an Area Under the Curve (AUC) of the UOC values of 0.94 and 0.91 respectively. This is followed by READv2 (AUC=0.89) and KIN (AUC=0.89) (Fig. 3B). TKGWV2 exhibited the lowest overall performance (AUC=0.82), despite showing good relative accuracy at lower coverage in the range [0.04–0.1]X. This is of course explained by the method’s incapacity to identify genetic relationships beyond the second degree. It should be noted that third-degree relationships can be misclassified by TKGWV2 with similar probabilities as either second-degree relationships or unrelated pairs (Fig. 3A). This inconsistent behaviour failed to be mitigated at higher coverages, and contrasts with the conduct of READ, which equally lacks the ability to detect third-degree relationships, but consistently reclassifies these pairs as unrelated (Additional file 2: Fig. S3). Likewise, READv2 implements a threshold of 5000 *expected* pairwise mismatches, below which the method will not attempt to classify third-degree relatives, instead preemptively assigning them to the unrelated class [25]. Note that this conservative threshold is directly derived from the median pairwise mismatch rate of the cohort, and began manifesting under these conditions at the 0.2X sequencing depth, corresponding to an average pairwise overlap of approximately *n*=29,000 SNPs (Fig. 3B, Additional file 1: Table S4). In line with the results obtained by Aktürk et al. [29], the classification performance of all methods plateaus at 0.2X, with the exception of KIN, whose performance significantly increases between 0.2 and 0.5X.

In general, a close inspection of the confusion matrices revealed two contrasting behaviours with regards to misclassifications (Fig. 3A and Additional file 2: Fig. S3A):

- Methods utilising median normalisation of PMR values such as KIN, READ and READv2, although generally exhibiting inferior accuracies across lower sequencing depths, are here characterised by a strikingly low amount of overpredictions (i.e. misclassifications lying above the main diagonal of the confusion matrix, that assign a degree of relatedness higher than the actual degree). This behaviour is retained at coverages as low as 0.04X, where the number of overpredictions merely reaches 17 pairs of individuals for READv2, and 25 for KIN over a total of 1300 predictions.
- On the contrary, methods using reference data such as correctKin, GRUPS-rs and TKGWV2 showcase a higher overall accuracy at lower sequencing depths. However, this higher performance comes at the price of a higher rate of overpredictions, with a total of *n*=83 overpredictions at 0.04X for correctKin, followed by *n*=129 for GRUPS-rs and *n*=181 for TKGWV2.

These two trends were also confirmed when investigating the normalised RMSD and MBE values for the *r*-coefficient estimation of every method, across all sequencing depths and degrees of relationship: KIN, READ and READv2 were generally less accurate and strongly biased in favour of underpredictions, while correctKin, GRUPS-rs and TKGWV2 on the other hand displayed lower nRMSD values and negligible bias (Additional file 2: Fig. S4). Regarding the performance of READ against its updated counterpart, READv2, these two methods showed little to no significant differences in their respective classification performance, apart from READv2’s distinguishing ability to detect third-degree relationships, which only started manifesting at a sequencing depth of 0.2X under the conditions simulated here (Additional file 2: Fig. S3). Given the similar behaviour of these two methods, we excluded READ from all subsequent investigations.

#### Impact of PMD correction methods

Next, we assessed the performance impact of employing either rescaling or masking as a PMD-correction strategy when preprocessing sample data. We have previously measured the performance of all methods using the default PMD-correction strategy of BADGER (Fig. 3), that is the in-house software pmd-mask (Additional file 2: Section 2). These initial results can be compared to the performances observed when alternatively using the PMD-rescaling software mapDamage2 [43], or the PMD-masking software trimBam [41].

**Fig. 4:**
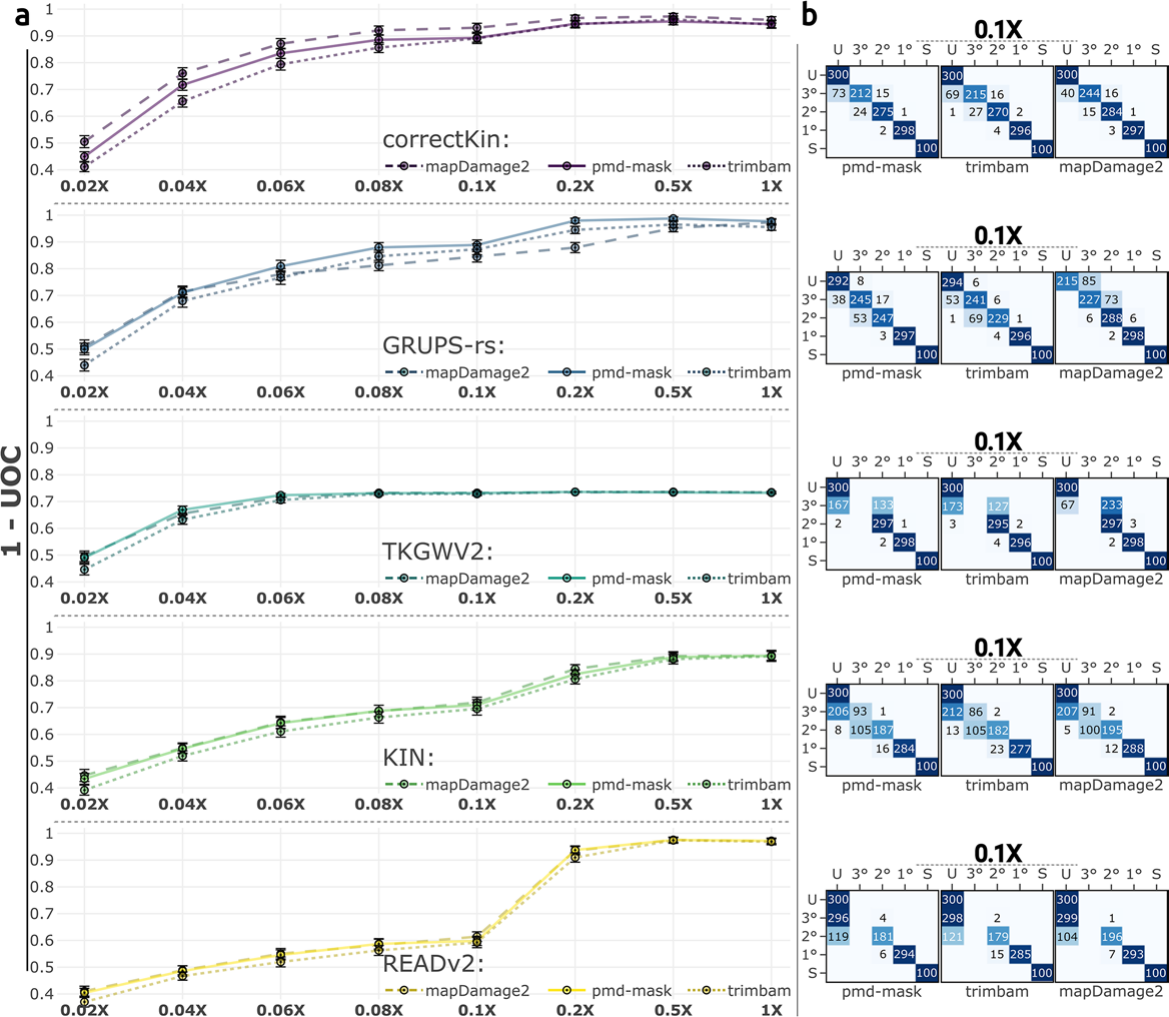
Evaluating classification performance against alternative PMD-correction strategies. **a** UOC values, summarising the classification performance of each method for the considered sequencing depths. Solid, dashed and dotted lines represent the performance resulting from the upstream application of the software pmd-mask, mapDamage2, and trimBam, respectively. Error bars represent 95% confidence intervals around the mean UOC values. **b** Selected subset of confusion matrices, at a set sequencing depth of 0.1X. Regarding mapDamage2 and trimBam, all confusion matrices for the considered parameter space are displayed in the supplementary materials (Additional file 2: Figs. S7 and S8)

For methods utilising reference data (correctKin, GRUPS-rs and TKGWV2), the application of mapDamage2 translated into an increased tendency towards higher *r*-coefficient estimates, compared with the application of pmd-mask and trimBam (Fig. 4, Additional file 2: Figs. S4, S5 and S6). For correctKin in particular, this increased positive bias proved beneficial under the particular conditions we are simulating, by improving the sensitivity of the method in delineating third-degree relationships (Fig. 4A and B). However, the upstream application of mapDamage2 resulted in a general *decrease* of the classification performance of GRUPS-rs, explained by a positive bias in the estimated *r*-coefficient, and resulting in an increase in misclassifications for unrelated and third-degree relationships, relatively to the application of pmd-mask. Though this effect was not sufficient to translate into lower $1-UOC$ values, this tendency towards overpredictions is equally displayed by TKGWV2, for which the number of third- to second-degree misclassifications increased from 133 to 233 when using mapDamage2 (Fig. 4B). On the other hand, the classification performance, mean bias and accuracy of KIN and READv2 remained unaffected by the upstream use of mapDamage2, in comparison with the application of pmd-mask, implying that the use of median PMR normalisation across samples may completely and adequately mitigate any bias brought about by the alteration of genotype frequencies resulting from PMD rescaling methods (Fig. 4, Additional file 2: Figs. S4 and S5). However, these results once again caution against jointly evaluating and comparing samples processed through different pretreatments, as batch effects may still occur in this case.

With regards to trimBam in particular, this benchmark shows that the upstream use of this software consistently delivers lower classification performance when estimating genetic ties, as compared to the pmd-mask software (Fig. 4A). This comparatively lower performance is irrespective of the average sequencing depth or genetic relatedness estimation method being used, and appears to intensify as the average sequencing depth decreases, particularly at depths lower than or equal to 0.06X. At these lower depths, the level of classification performance obtained when using trimBam is consistently the lowest out of the three PMD-correction methods. This general pattern likely stems from the fact that the masking strategy devised in trimBam can cause significant data loss – one that is expected to be stronger than that incurred by pmd-mask – ultimately resulting in a significantly lower average pairwise SNP overlap, when assessing genetic ties.

However, when greater average sequencing depths are considered, trimBam can potentially yield higher overall performance than mapDamage2. This is most evident in the case of GRUPS-rs at depths greater than 0.06X, where trimBam maintains performance levels halfway between pmd-mask and mapDamage2. At these higher sequencing depths, the data loss induced by trimBam therefore appears to have a lesser impact than the biased *r*-coefficients observed under the mapDamage2 condition (Fig. 4B, Additional file 2: Figs. S5 and S6).

#### Impact of modern human contamination

**Fig. 5:**
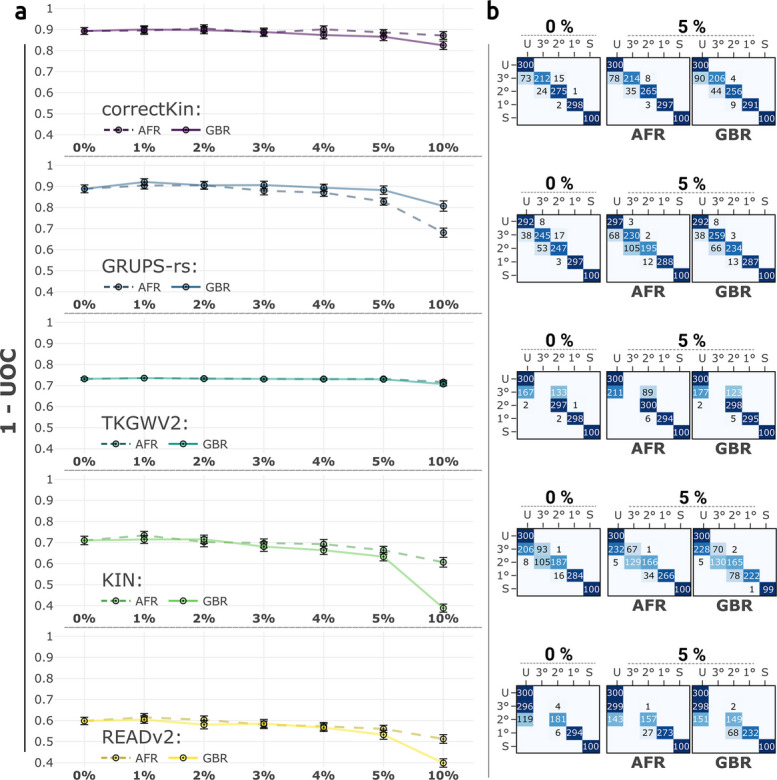
Evaluating performance against increasing rates of modern human contamination. **a** UOC values summarising the classification performance of each method for the considered rates of contamination. Solid and dashed lines represent the performance resulting from randomly sampling contaminating individuals from the GBR and AFR populations, respectively. Error bars represent 95% confidence intervals around the mean UOC values. **b** Selected subset of confusion matrices, at a set contamination rate of 5%. Complete displays of all confusion matrices for the AFR and GBR conditions, across all the parameter space may be seen in the supplementary materials (Additional file 2: Figs. S11 and S12)

Next, the impact of modern human contamination was evaluated at a constant sequencing depth of 0.1X, by incorporating between 0 and 10% of exogenous sequences from a single randomly sampled individual, belonging either to the African [AFR] super-population, or to the European British [GBR] population, which is comparatively more closely related to the source population of the pedigree individuals [CEU] (Fig. 5). In general, these results highlight that contamination has a negligible impact when below 5% (Fig. 5A). The addition of sequences stemming from a single contaminating individual led to an increased *negative* bias and variance of the *r*-coefficient estimates produced by every method, resulting in the production of underpredictions (Fig. 5B, Additional file 2: Figs. S9 and S10). Moreover, the genetic proximity between the contaminating individual and the contaminated samples appears to significantly influence the classification performance at rates equal to or greater than 5%. Thus, all methods apart from GRUPS-rs displayed a lower performance when contamination stemmed from a GBR individual (hence, belonging to the same super-population as the individuals from the simulated pedigree) than from an AFR individual.

Here, this distinguishing behaviour of GRUPS-rs can be partially explained by the fact that this method inherently compares the average heterozygosity between a predefined, user-selected reference panel of genotypes – in this case, individuals from the EUR super-population [32] – and the samples being studied [24]: thus, contamination stemming from a different population or super-population, as showcased in the simulated AFR condition (Fig. 5A), is expected to produce a higher rate of divergence between the tested samples and the reference panel.

#### Impact of population diversity and reference panel bias

**Fig. 6:**
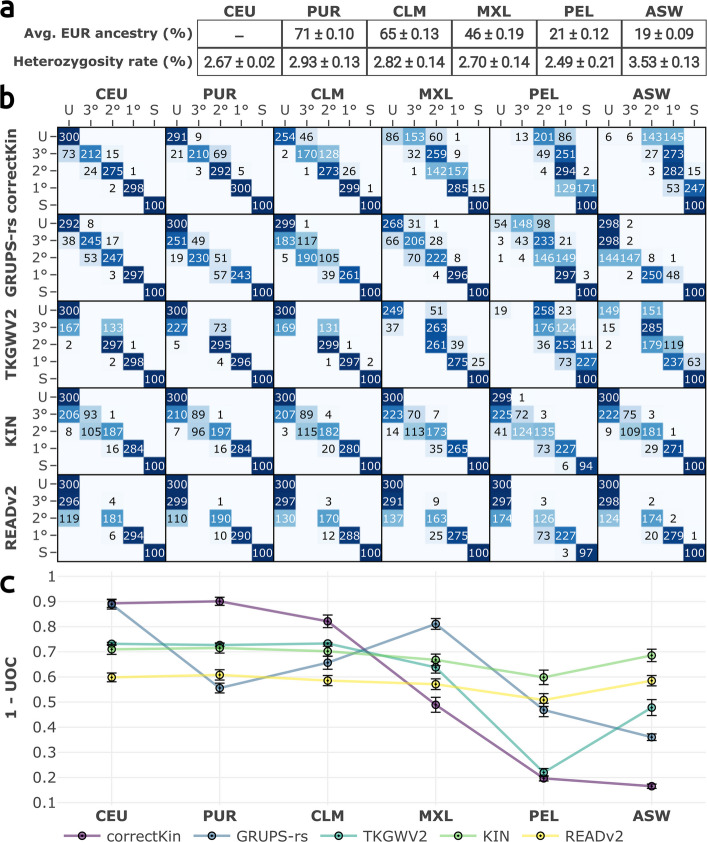
Evaluating the impact of genetic diversity and reference panel bias on classification performance, at a fixed sequencing depth 0.1X. **a** Average European ancestry, and heterozygosity proportions ($\pm \sigma$) of every tested population. Proportions of EUR ancestry are calculated from the results of [47]. Complete distributions of local ancestry and heterozygosity rates of all AMR populations may be visualised in Additional file 2: Figs. S13 and S14, respectively. **b** Confusion matrices confronting expected and predicted relationships. **c** UOC values summarising the classification performance of each method for the considered source population. Error bars represent 95% confidence intervals around the mean UOC values

Next, we reapplied BADGER simulations at a set sequencing depth of 0.1X, alternately selecting the PUR, CLM, MXL, PEL and ASW populations as the source of founder individuals (Fig. 6) within the simulated pedigrees. These five admixed American populations (AMR) are known to carry varying levels of European ancestry, as well as differing patterns of heterozygosity rate distributions (Fig. 6A, Additional file 2: Figs. S13 and S14) [32, 47].

First, the classification performance of KIN and READv2 (i.e. methods utilising PMR normalisation of samples, and thus lacking any input reference data), remained generally unaffected when applied against the PUR, CLM and ASW populations. However, a stronger spread and bias of *r*-coefficients is observed with the MXL and PEL populations, and thus a decline of performance (Fig. 6B and C, Additional file 2: Fig. S15).

Contrastingly, the results displayed by correctKin, GRUPS-rs and TKGWV2 highlight that providing these methods with reference data deviating from the source population of the pedigree individuals can drastically impact their performance (Fig. 6C). This plummet of performance is seen most strongly with correctKin, which exhibited a 9-fold decrease of its UOC performance values, between the CEU and ASW conditions, resulting in an overall accuracy of merely 12.5%. Additional simulations involving the ASW population, conducted across the entire sequencing depth range [0.02 – 1]X, showed that higher sample coverage failed to mitigate this bias, even at a sequencing depth of 1X (Additional file 2: Fig. S16).

Here, it must be noted that the decreased performance exhibited by these five methods is expressed through different patterns:

- For correctKin, the decrease in performance largely appears to follow the level of shared ancestry between the reference population provided to the software (in this case, individuals from the EUR super-population), and the source population used in the pedigree simulations of BADGER [48]. Thus, lower shared ancestry levels generally produce a stronger positive bias, and an increased spread in the *r*-coefficient estimates (Fig. 6A and B, Additional file 2: Fig. S15).
- For GRUPS-rs, the decrease in performance and increase in bias do *not* display this pattern, but rather correlate with the difference between the average heterozygosity rate of the input reference panel (i.e., 1000g-phase3 EUR genotypes) and of the selected source population for the pedigree simulations [48]. Negative bias and the spread of *r*-coefficient estimates thus increase as the heterozygosity rate of the source population for the pedigree simulation goes higher than that of the provided reference panel (Fig. 6A and B, Additional file 2: Fig. S15). However, the method may instead become *positively* biased, in cases where the heterozygosity rate of the tested population is *lower* than that of the reference population, as exemplified with the PEL condition (Fig. 6B).
- For KIN and READv2, the performance pattern predominantly follows not simply the heterozygosity rate of the tested population, but more precisely its relative standard deviation (i.e.: $RSD = \sigma / \mu$), which is found highest in the case of the PEL population (Fig. 6A). (For clarity, note that ordering these six populations according to this metric returns $CEU< ASW< PUR< CLM< MXL < PEL$.)
- TKGWV2 here exhibits a “mixed” pattern, which cannot be fully explained solely by the heterozygosity rate or level of shared ancestry. This behaviour can be partially explained by the fact that this method is directly reliant on input reference allele frequencies, and that the Queller and Goodnight estimator used within TKGWV2 was previously demonstrated to be either positively or negatively biased when the provided allele frequencies are biased [34]).

#### Impact of inbreeding

**Fig. 7:**
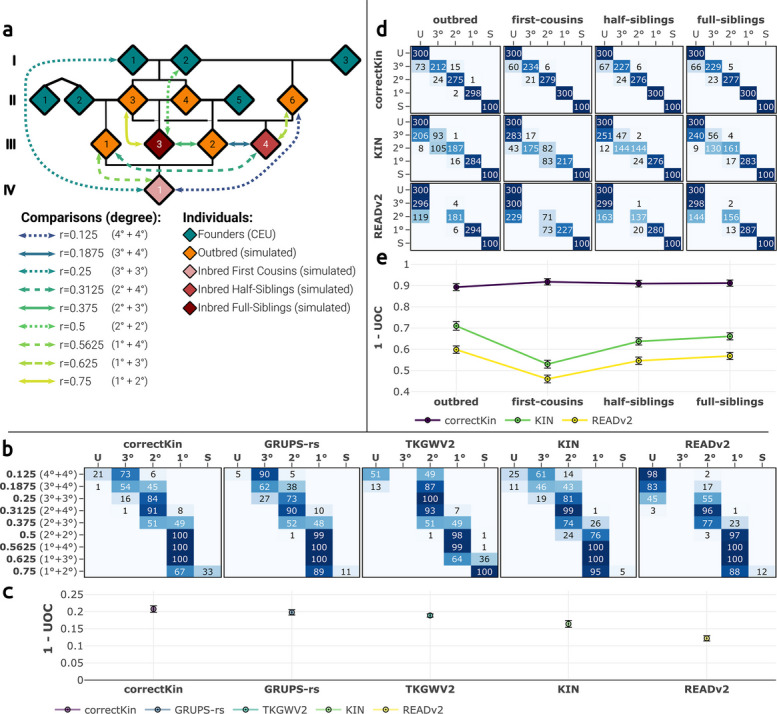
Evaluating the impact of close inbreeding on classification performance. **a** Alternative template pedigree provided to BADGER when evaluating the impact of close inbreeding. Coloured arrows denote the pairwise relationships investigated during the inbreeding benchmark. Additional inbred individuals are coloured in shades of red, with individuals III.3, III.4 and IV.1 being respectively the result of a mating involving two full-siblings, two half-siblings, and two first-cousins. **b** Confusion matrices confronting expected and predicted relationships involving an inbred individual. **c** UOC values summarising the classification performance of each method, for the considered scenarii of (**b**). Error bars represent 95% confidence intervals around the mean UOC values. **d** Confusion matrices confronting expected and predicted relationships of outbred relationships for correctKin, KIN and READv2. **e** UOC values summarising the classification of each method, for the considered scenarii of (**d**)

Next, we assessed the impact of the presence of closely inbred individuals among the studied samples. To this end, we ran 100 simulations replicates of BADGER at a sequencing depth of 0.1X, using an alternative pedigree containing a total of three inbred individuals (Fig. 7A).

For comparisons involving inbred individuals (Fig. 7A-C), the behaviour of each method here generally followed the same trends observed in the analysis of outbred individuals – all things being proportionate to the fact that, by construction, none of these methods is able to distinguish between intermediate degrees of relatedness. Hence, the classification of $4^{\circ }+4^{\circ }$, $3^{\circ }+3^{\circ }$ and $2^{\circ }+2^{\circ }$ relationships was predominantly accurate, and these cases were categorised as third-, second- and first-degree relationships, respectively. (Note that sharing several genetic ties with another individual has a *compounding* effect on the resulting degree of relatedness: for example, double first-cousins are linked through *two* third-degree ties ($3^{\circ }+3^{\circ }$), and are thus expected to display an *r*-coefficient akin to a *second-degree* relationship.) Comparing the sensitivity of these comparisons against the outbred scenario showed no significant differences for all methods, apart from KIN, which exhibited a higher number of misclassifications when investigating these specific inbred relationships (Figs. 3A, 7B, Additional file 1: Table S5). This performance drop was also reflected by the nRMSD and nMBE values (Additional file 2: Fig. S17), where KIN in this particular case reached the highest dispersion and bias rates at relatedness coefficients greater than or equal to 0.375, whereas this method showed high relative precision when only exposed to outbred comparisons (Additional file 2: Fig. S4). Intermediate degrees of relationships are on the other hand generally classified according to the strongest degree that is shared between the pair. Hence, $1^{\circ }+4^{\circ }$, and $1^{\circ }+3^{\circ }$ are predominantly classified as first-degree relationships, and $2^{\circ }+4^{\circ }$, as second-degree. Unsurprisingly, $1^{\circ }+2^{\circ }$, $2^{\circ }+3^{\circ }$ and $3^{\circ }+4^{\circ }$ relationships displayed the highest rate of misclassifications, as their respective expected relatedness coefficient are located at the *midpoint* between two prediction classes. Overall, the relative order of the classification performance of each method here mirrors that of the outbred scenario. Hence, correctKin showed the best performance at this specific sequencing depth (0.1X), followed by GRUPS-rs, TKGWV2, KIN, and finally READv2 (Figs. 7C, 3B).

While TKGWV2 and GRUPS-rs operate independently on every pairwise comparison, READv2, KIN and correctKin all apply a *joint* estimation of genetic relatedness, on the supplied cohort. As such, the inclusion of an additional inbred individual, and its genetic ties with the rest of the cohort may potentially influence the normalisation procedure of these methods. For this reason, we evaluated the impact of this specific interaction on the performance of KIN, READv2 and correctKin, alternatively testing the three inbreeding scenarii, and focusing this time on the effect incurred on *outbred* relationships (Fig. 7D and E). This analysis revealed that the presence of inbred individuals can strongly affect the normalisation procedure of READv2 and KIN, as evidenced by an increase of underpredictions (Fig. 7D and E, Additional file 2: Fig. S18). This performance decline cannot be solely explained by the greater propensity of inbred individuals on reducing the overall genetic diversity of the cohort. Nor can it be directly anticipated from the level of inbreeding of the added individual, given that the largest performance decline occurs in the “first-cousins” scenario (Fig. 7E). Given the limited sample size of our three scenarii, the specific *position* of the inbred individual within the tree’s topology may be a contributing factor to the observed performance pattern. Indeed, out of a total of 66 comparisons, the pedigree provided for the “half-siblings” and “full-siblings” scenarii respectively contains 34 and 35 unrelated pairs, suggesting that the median of comparisons calculated by READv2 and KIN is still statistically expected to fall within the bounds of an unrelated comparison (Additional file 2: Fig. S2). In contrast, the “first-cousins” scenario merely contains 31 unrelated pairs, for an equal sample size, resulting in a median that will statistically fall within the bounds of a third-degree comparison.

## Discussion

Drawing upon the results obtained using BADGER, several overarching observations may be deduced from our proposed benchmark parameter space. Firstly, the classification performance of methods utilising reference data reaches a stabilisation point at an average sequencing depth of 0.2X (Fig. 3B), which in the particular conditions simulated here represents an average pairwise overlap of around 28*k* SNPs (Additional file 1: Table S4). These results are in line with the previous findings of Aktürk et al. [29], who equally reported consistent performance among all tested methods when the average pairwise overlap was $\ge 20k$ SNPs. However, these conclusions are not strictly applicable to KIN and READv2, which, given the limited sample size of the provided pedigree, only reach this performance equilibrium at 0.5X. Yet, despite their limited ability to delineate third-degree relationships, the performance behaviour of these two cohort-normalisation methods may still prove desirable, even at such small sample size, considering their general lack of overpredictions (Fig. 3), and robustness against contamination, batch effects resulting from the upstream use of PMD-correction software, or presence of admixture within the tested population (Figs. 4B, 5B, and 6C). However, it should be emphasised that the overall inclination of READ, READv2 and KIN towards underpredictions reported in the current study stands in contrast to previous findings by Marsh et al. [28] and Aktürk et al. [29], which showed a comparatively higher number of overpredictions for these three methods, due to their lower accuracy in estimating *r*-coefficients [28, 29]. This previously unreported “conservativeness” is likely due to the fact that the benchmark design of the present study simulates the application of these three methods on *small* cohorts of closely related individuals (As a reminder: $n = 55$ pairwise comparisons, for only 31 pairs of unrelated individuals). Therefore, the results obtained from this study suggest that the application of these three methods on very large cohorts and/or the inclusion of additional unrelated individuals from other “proxy” populations as a means to artificially increase the size of the analysed cohort — an approach occasionally employed in the current literature (e.g.: [5]) — could prove counter-productive, and increase the risk of reporting false positives.

Thus, for most intents and purposes, and provided a sufficient sample size and/or adequate reference data, an average pairwise overlap of 28000 SNPs across all samples should be regarded as a conservative prerequisite for the full implementation of all of these methods, thereby ensuring precise delineation of second- and third-degree relationships. For the specific delineation of first-degree relationships, monozygotic twins and duplicate samples, however, sequencing depths as low as 0.04X, corresponding to around 1400 pairwise overlapping SNPs, may be used with fair confidence (Table 2, Additional file 1: Table S4).

**Table 2:** Minimal average sequencing depth required to achieve $F1-score> 0.95$ across all discernible degrees of relatedness

Degree	correctKin	GRUPS-rs	TKGWV2	KIN	READv2
*Unrelated*	–	0.2X	–	–	0.2X
$3^{rd} degree$	–	0.2X	–	–	0.5X
$2^{nd} degree$	0.2X	0.2X	–	0.2X	0.5X
$1^{st} degree$	0.06X	0.06X	0.06X	0.1X	0.08X
$Monozygotic\ twins$	0.04X	0.04X	0.06X	0.04X	0.04X

One-vs-all $F1-scores$ were calculated using the caret package [46], and are defined as the harmonic mean of the precision and recall of a given class ($F1= 2\frac{precision \cdot recall}{precision+recall}$). Per-class $F1-scores$ across the entire benchmark parameter space, as well as several other nominal performance metrics are exhaustively reported in Additional file 1: Table S3. “–” implies that the 0.95 threshold was never reached by the method, for the considered degree of relationship

Regarding correctKin and GRUPS-rs, these methods displayed the highest overall performance, most likely due to their use of reference data (Fig. 3). However, this study highlights that this apparent superiority is strongly contingent upon the quality of the data upon which it is applied, and may be strongly sensitive to biased reference datasets, batch effects, or upstream data processing (Figs. 4 and 6). Of note, the interpretation of these latter findings should not be confined to pseudo-haploidisation methods, but rather corroborates and supplements those reported by Aktürk et al. [29] and Marsh et al. [28] regarding NgsRelate and lcMLkin. These two methods, despite their use of genotype likelihoods, also exhibited subpar performance when applied against “noisy” population allele frequencies – i.e. where random fluctuations around the measured allele frequencies are introduced through varying levels of gaussian noise [29]. This general issue regarding methods utilising reference data is of strong significance and should not be overlooked when studying populations originating from distant pasts, for which genetic drift, and lack of any accurate reference dataset may take significant effect. Moreover, we strongly suspect and caution that this sensitivity to batch effects and reference panel bias, here specifically exhibited by correctKin, GRUPS-rs and TKGWV2, should also be considered when applying READv2 and KIN along with a *fixed* user-provided normalisation value and/or when including additional “proxy” individuals within the cohort in order to compensate for an insufficient sample size.

Likewise, the evidence presented here for a deleterious impact of mapDamage2 on TKGWV2 and GRUPS-rs (Fig. 4) complements and feeds into recent findings of Koptekin et al. [42], who similarly reported significant reference bias when applying this PMD-rescaling method [42]. While this bias proved *beneficial* for correctKin under the conditions simulated here (Fig. 4), these results still imply that correctKin may be sensitive to batch effects, and hint at the larger issue of comparing individuals processed through differing methods, which could exacerbate the risk of generating false positive results. Taken altogether, these results compel us to advise against the use of PMD-rescaling methods for proper genetic relatedness estimation, and to instead turn to alternatives such as pmd-mask, or the software bamRefine [42], the latter being specifically designed to address this issue, and demonstrated to be a good compromise between mitigating reference bias, data loss and *post-mortem* damage [42].

Our assessment of the impact of inbreeding on these methods demonstrated no discernible systematic bias shared amongst all methods, suggesting that its presence is scarcely detectable in real use-case scenarii without a closer inspection of the raw *r*-coefficient estimates. Due to their reliance on a cohort-normalisation procedure, KIN and READv2 here suffer a double penalty which is inversely correlated with the provided sample size, since the presence of inbred individuals could first deviate the median PMR calculated during the normalisation step, which would in turn cause the method to provide the end-user with negatively biased *r*-coefficient estimates, making the detection of this bias more challenging. Intermediate relationships such as e.g. three-quarter siblings are in any case not considered during the classification procedure of these methods. While confirmed cases of close incest remain sparse [9], the fact that most of these methods fail to fully capture this signal remains of significance, considering that complex marital practices (e.g. levirate/sororate unions, double-cousins, etc.), though not strictly incestual, may also generate intermediate degrees of relationships, and are known to be practised throughout ancient cultures [1, 7, 49]. For this reason, the systematic use of complementary methods designed to detect signals of inbreeding is here highly recommended when attempting to estimate genetic relatedness [49, 50], when applicable. Likewise, GRUPS-rs’ distinctive ability to formulate user-defined scenarii of genetic relatedness, or the capacity of KIN to detect long runs of homozygosity (ROH) are equally of relevance, although their respective effectiveness was not assessed during this benchmark.

On the effect of contamination, the results of this study broadly suggest that it exerts negligible influence at proportions below 5%. However, these should be acknowledged as an optimistic measure, given that they only reflect a specific condition (i.e. originating from a single person, and distributed equally across all samples). In other terms, these findings should by no means be generalised to all possible scenarii and cannot *de facto* be extended to cases where e.g. several sources of contamination are expected and/or where the rate of contamination differs between the samples tested. Furthermore, this study did not take into account the contamination correction procedures available in the algorithms of KIN and GRUPS-rs [21, 24], although BADGER is able to parametrise these two methods accordingly. Their overall effectiveness in mitigating this specific bias is therefore potentially underestimated.

In light of the demonstrably unpredictable behaviour exhibited by some of these methods, and the number of variables required to fully model all of the biological parameters associated with ancient DNA sequencing data, we posit that the repurposing of BADGER in real-world scenarii could prove useful. Specifically, BADGER may be regarded as a framework for simulating, on a case-by-case basis, the *expected* behaviour of several genetic relatedness estimation methods, based on the genetic data available to the user. Though computationally intensive, this approach has the potential to facilitate the interpretation of results obtained through these methods, in cases that are challenging to resolve due to the presence of low-quality data, or when the reliability of the results is deemed to be low. In this regard, the development of BADGER has been consistently pursued with the objective of enhancing its reusability, and ensuring that its interface remains intuitive and user-friendly. Thus, the entire BADGER pipeline can be interacted with a single, fully documented command line interface, and merely requires the preparation of a single YAML configuration file, to provide the software with simulation parameters (e.g. average sequencing depth, rate of contamination, fragment length frequency distribution, PMD-damage parameters, etc.). In a similar vein, BADGER is bundled with a documented and command-line interfaced R library (“badger.plots”), thereby enabling users to automatically generate figures, similar to those presented in this manuscript, which summarise the performance of the methods benchmarked through BADGER. In its current state, BADGER offers native support for the joint benchmark of the software correctKin, GRUPS-rs, READ, READv2, KIN and TKGWV2. However, the software can feasibly be extended with additional target software to benchmark, provided common knowledge of the snakemake domain specific language [35].

### Current limitations

#### Use of modern reference data

The benchmark presented here currently makes consistent use of modern data, both as a source of founder individuals during the pedigree simulations and as a reference data for the benchmarked methods. This design choice is advantageous in that it provides readers and investigators with reliable information regarding the level of heterozygosity and shared ancestry between these two populations, thereby facilitating the interpretation of the results presented in Section Impact of population diversity and reference panel bias. However, this approach also carries the disadvantage that the simulations devised here *never* take into account any bias potentially introduced by the effect of genetic drift, background relatedness or ascertainment bias. While BADGER can introduce slight ascertainment bias by e.g. using a reference dataset which constitutes a superset of the source population of founder individuals (as it has been done during this benchmark, by using the EUR superpopulation as the reference data, versus the CEU population as the source for founder individuals), the software is currently devoid of any procedure allowing for the simulation of genetic drift, or recent population bottlenecks.

Conversely, the results presented in Section Impact of population diversity and reference panel bias have shown that *any* genetic discrepancy between the individuals tested and the reference data used can significantly impact the performance of the three methods using reference data (correctKin, GRUPS-rs and TKGWV2). Taken together, these two points imply that the performance exhibited by correctKin, GRUPS-rs and TKGWV2 in this study is likely optimistic, particularly when considering samples that are chronologically distant, poorly referenced and/or from populations expected to have a small effective population size. Given the results presented in Section Impact of population diversity and reference panel bias, we therefore must emphasise that it is generally advisable, whenever feasible, to always verify the absence of population structure within the sample being tested, and to ensure that the levels of common ancestry and heterozygosity between ancient samples and reference data are adequately matched, as these insights could help the analyst to better understand the expected performance of these methods before applying them.

#### Translating these results for sequence capture data

Similarly, the benchmark results presented here can only be directly applied to data obtained by shotgun sequencing. This limitation is partly due to a restriction in the software used to generate raw fragments within BADGER (gargammel), but it is also the result of a deliberate design choice, motivated by the growing number of shotgun-sequenced ancient samples, and previous reports of allelic bias related to the use of in-solution capture data [42, 51].

Nevertheless, this study can still provide some key insights into these specific data. Firstly, such protocols would likely decrease the overall level of heterozygosity in the ancient samples tested due to the potential increase in allelic bias. This particular point could certainly represent a source of bias for methods such as GRUPS-rs, READv2 and KIN, and was already reported in the specific case of READ [51]. Similarly, allelic bias is expected to negatively impact TKGWV2, given that this method uses allele frequencies as a reference point. Conversely, the few methods tested which calculate a form of long-term average of the PMR, namely KIN and GRUPS-rs, could still benefit from the local increase in sequencing depth generally yielded from the use of capture protocols. However, the gain-to-loss ratio between the effect incurred by the increased local sequencing depth and that of allelic bias for these two methods remains difficult to anticipate precisely without further analysis. Likewise, the expected behaviour of correctKin is hard to delineate without further analyses, given that this method internally makes use of principal component analysis. However, considering that this latter method exhibited a higher performance when applied against PMD-rescaled data (which is also most likely a source of reference bias [42]), an apparent increase of performance could be expected once applied to capture data, as it would help this method in delineating ancient individuals from the modern reference data.

## Conclusions

Despite recent methodological advances, this study demonstrates that the performance profile of many currently available genetic relatedness estimation methods cannot be predicted from pairwise sample SNP overlap alone, and may succumb to other sources of bias, particularly from the use of poorly ascertained reference datasets. Given the complementarity of these two sub-approaches, we therefore systematically recommend the combined use and cross-checking of the results provided by at least one method using reference datasets (correctKin or GRUPS-rs), and at least one non-parametric method leveraging PMR normalisation (READv2 or KIN). This study also introduces the BADGER software, which has not only facilitated the benchmark conducted here, but also represents an additional user-configurable method, which can be leveraged by users to better anticipate the performance and behaviour of these different statistical tools, according to their specific use case.

## Methods

### Description of BADGER’s simulation pipeline

#### 1000 genomes dataset pre-processing

BADGER first downloads the 1000 genomes phase3-v5b dataset from the EMBL–EBI FTP website [32] (Additional file 2: Section “Key Resources Table”) and proceeds to apply normalisation and left-alignment of indels, using bcftools norm [52]. Multi-allelic positions and any lingering unphased genotypes are then filtered out using bcftools view (–-phased -m2 -M2). From the processed dataset, BADGER then generates two data subsets according to the population or superpopulation label of the samples:

- A concatenated VCF file containing all samples belonging to the selected founder population. This file is used as an input to Ped-sim when simulating pedigrees, as a source of founder individuals. During this study, the selected founder population was either CEU, to simulate a genetically homogeneous population, or one of the five populations belonging to the admixed American population (ASW, CLM, MXL, PEL, PUR) to simulate an admixed population.
- A set of VCF files (one for each autosome), containing all samples belonging to the selected contaminating population is generated. This second dataset is used as input for gargammel when simulating ancient DNA fragments to extract a single contaminating individual, and use its genotype as a source of modern human contamination. During this study, we selected either the AFR superpopulation, or the GBR population as a source to simulate modern human contamination.

#### Pedigree simulations

BADGER leverages the software Ped-sim to simulate pedigrees in multiple replicates, using founder individuals randomly selected from the founder dataset. Here, we parametrised Ped-sim to simulate sex-specific recombination rates, as well as a crossover interference model, using the refined genetic map from [53] and the interference parameter estimates of [54], respectively. To maximise the number of possible combinations, and given that BADGER only simulates autosomes, the original genetic sex of the individuals selected as founders was not taken into account to select founders within the pedigree replicates. Simulation of genotyping errors, opposite homozygous errors, missingness, and pseudo-haploid rates were all disabled at this step, to prevent any compounding interactions with the error model of gargammel (–-err_rate 0 –-err_hom_rate 0 –-miss_rate 0 –-pseudo_hap 0).

Simulation of monozygotic twins and/or duplicate samples within the pedigree is performed by merely duplicating the genotype of the selected individuals within the output VCF of Ped-sim (see rule “$dopplegang\_twins$”, Additional file 2: Fig. S19).

#### Ancient DNA simulations

Simulation of raw ancient DNA fragments for every pedigree individual is performed using gargammel. As this software requires the use of FASTA-format haplotype sequences, BADGER first uses bcftools consensus to apply the variants from the output VCF file of Ped-sim to a reference fasta file, thus generating a consensus sequence for every pedigree individual and, when simulating non-null rates of modern human contamination, a randomly selected contaminating sample from the contaminant dataset.

Here, note that copy-number variations, two-sided inversions, and insertions of ALU, LINE1, SVA and Nuclear Mitochondrial elements are filtered out using regular expressions, to comply with the requirements of bcftools (–-exclude ’ALT ”<CN[0–9].*>”||ALT ”<INS:.*>” || ALT ”<INV>’).

For every individual, haplotype sequences are then inserted in the required “endo” input directory of gargammel. Likewise, when simulating human contamination, the haplotype sequences of the randomly sampled individual are inserted in the optional “cont” input directory. BADGER then applies gargammel on these input directories, using the user-provided misincorporation probability and fragment size frequency profiles. Here, we elected to use the *post-mortem* damage profile of “Chan_Meso”: a young adult female individual dated from the Mesolithic period ($9137 \pm 124 \, cal.\ BP$) and exhumed from the “Chan do Lindero” karst system of Pedrafita, Lugo, Spain [55]. This choice of reference was motivated by the fact that Chan_Meso was sequenced on an Illumina HiSeq2000 platform – one of the preset sequencing platform model choice for gargammel’s – and exhibits “average” *post-mortem* damage patterns (i.e. an approximate misincorporation rate of 0.22, at the $3'$- and $5'$-end of reads, and a mode of approximately 70 base pairs on its fragment size frequency distribution). Note that while BADGER can be parametrised to handle bacterial contamination from publicly available databases, this capacity was not leveraged during the present study.

To optimise the I/O throughput and runtime performance of BADGER, generation of ancient DNA fragments using gargammel is applied in parallel for every pedigree individual, on a per-chromosome basis, using a simple scatter-gather approach. Hence, per-chromosome FASTQ files are merely concatenated using the zcat and gzip UNIX command-line utilities.

#### Alignment

The raw paired-end fragments of every individual composing the pedigree are then trimmed of adapter sequences and collapsed, using AdapterRemoval v2 [56], requiring a minimum adapter overlap of 1, read length of 17 and base quality of 20. (–-minlength 17 –-minquality 20 –-minadapteroverlap 1).

Trimmed fragments are then aligned against the GRCh37 reference genome [57], using bwa aln [58], following the best practices described in [39] (-l 1024 -n 0.01 -k 2 -o 2). Note that collapsed single-end sequences and non-collapsed paired-end sequences are mapped separately, using bwa samse and bwa sampe respectively, and then merged using samtools merge. Here, a generic read group tag is placed using samtools addreplacerg following merging.

#### Quality filtering and preprocessing of alignment files

Following alignment and merging, a simple quality filtration step is first applied to the raw binary alignment files of every sample using samtools view. Hence, the raw files are trimmed of any sequence that is either a) unmapped, b) measuring less than 30 nucleotides, or c) carrying a mapping quality score lower than 20 (PHRED scale) (-F4 -q20 -e ’length(seq)>30’).

Alignment files are then sorted using samtools sort and removed of any optical PCR duplicates using picard MarkDuplicates [59] (–-REMOVE_DUPLICATES true –-VALIDATION_STRINGENCY LENIENT –-ASSUME_SORT_ORDER coordinate)

#### Correction of *post-mortem* deaminations

Patterns of *post-mortem* deamination were estimated on every sample alignment file, using mapDamage2. To estimate the performance impact of applying *post-mortem* damage rescaling, three alternative post-processing methods were then applied:

- “Rescaled” versions of the alignment files, wherein the base quality scores of putative nucleotide misincorporation sites are downscaled, were generated by applying the –-rescale flag of mapDamage2.
- “Masked” versions of the alignment files were generated using the in-house software pmd-mask and the misincorporation probability estimates of mapDamage2 (misincorporation.txt file). Here, potential C>T and G>A deamination sites are masked all together, along the 5’ and 3’ ends of fragments, respectively, until the misincorporation probability is less than 1%.
- “Soft-clipped” versions of the alignment files were obtained using module trimBam of the bamUtil software. Here, a user-defined length threshold (in base pairs) is used to *exhaustively* mask (a.k.a soft-clipping) the 3’ and 5’ of every read. For all conditions shown during this benchmark, we applied a set threshold of 10 base pairs, to replace all nucleotides as ’N’ along the end of each read (i.e. –-left 10 –-right 10).

#### Variant calling

Next, BADGER jointly applies random pseudo-haploid variant calling on every post-processed alignment file by first creating a pileup file with samtools mpileup. Here, autosomal bi-allelic SNP positions from the AADR “1240K” SNP dataset, version 52.2 were targeted [60], while disabling Base alignment quality (BAQ) recalculation, and filtering out any position with a mapping and/or base quality lower than 20 (-RB -q20 -Q20). This pileup file is then directly given as input to the pileupCaller module of sequenceTools [61], to generate pseudo-haploid variant calls (–-randomHaploid –-minDepth 1), in binary PLINK format. The use of sequenceTools pileupCaller is a default setting that is applied consistently throughout this benchmark. However, note that BADGER also optionally allows variant calling to be performed using the doHaploCall module of the ANGSD software suite [62].

#### Genetic relatedness estimation

Note that the benchmarked genetic relatedness estimation methods may have differing input data. Hence:

- The random pseudo-haploid variant calls of pileupCaller were given as input to READ and READv2.
- The joint pileup file of samtools mpileup was given as input to GRUPS-rs.
- The post-processed binary alignment files of every sample composing a pedigree replicate were given as input to correctKin, KIN and TKGWV2.

##### correctKin

Following the guidelines of [22], BADGER first generates a subset of the AADR “1240K” dataset [60], to provide correctKin with a user-selected set of reference individual genotypes. Here, all samples belonging to the EUR superpopulation of the 1000g-phase3 dataset and contained within the 1240K dataset were selected as reference individuals for correctKin during this study. However, as BADGER also makes use of 1000-genomes samples as a source of founder individuals during pedigree simulations, the pipeline first excludes any sample previously given as an input to Ped-sim, from the list of reference samples added to the correctKin input dataset. BADGER then merges the resulting “1240K” data subset with the pseudo-haploid variant callset of the pedigree replicate, using plink [63] (–-bmerge –-merge-mode 1 –-allow-no-sex –-keep-allele-order). Still following guidelines, a covariance matrix and a marker overlap fraction matrix are generated from this merged dataset, using pcangsd [64] and the markerOverlap module of correctKin. Unrelated individuals were filtered out using the filterRelates module of correctKin. Here, note that, a) all pairs of individuals not found in the resulting output file were considered as unrelated, and b) pairs of individuals classified as “uncertain” are reclassified as unrelated.

##### GRUPS-rs

Following the guidelines of [24], BADGER first creates an FSA-encoded dataset of reference individuals from the raw 1000-genomes phase3 dataset, using the grups-rs fst module. This preprocessing step is merely intended to increase the runtime efficiency of BADGER and the resulting fsa-encoded 1000g-phase3 dataset is used as an input throughout all pedigree replicates. For every pedigree replicate, BADGER then directly applies grups-rs pedigree-sims on the pileup file described in Section Variant calling, while requesting 1000 simulation replicates, and using samples from the 1000g-phase3 EUR superpopulation as reference individuals (–-reps 1000 –-pedigree-pop EUR –-min-depth 1 –-seq-error-rate 0.0). Note that recombination maps from the HapMap-II dataset are also provided to GRUPS-rs at this stage, in order to directly calculate crossover probabilities during the software’s internal pedigree simulations [65].

Since GRUPS-rs requires the use of a user-constructed template pedigree to perform its simulations, we provided the software with the same template throughout this study (Additional file 2: Fig. S20). Note that this simple pre-constructed template is made available as an example within the sofware’s documentation, contains a pair of siblings, half-siblings and first-cousins, and uses these comparisons to estimate first-, second- and third-degree relationships, respectively.

##### KIN

BADGER first applies the KINgaroo module on the post-processed binary alignment files of all individuals composing a pedigree replicate. Bi-allelic autosomal SNPs from the “1240K” dataset are targeted during this preprocessing step (–-bedfile), while disabling contamination correction (–-contam_parameter 0). The main KIN module is then applied on the output of KINgaroo using default parameters. From the final output of KIN, a coefficient of relatedness *r* is deduced for all tested pairs of individuals, using the provided Cotterman coefficient estimates for every given pair, i.e.: $r = k_{1}/2 + k_{2}$.

##### READ

The pseudo-haploid variant callset of pileupCaller described in Section Variant calling is given as input to READ, with default arguments (normalisation statistic: median, sliding window size: $10^6$ bp).

From the final output of READ, the relatedness coefficient *r* of every tested pair of individuals is derived from the normalised $\overline{P}_{0}$ estimates of a given pair, using the following equation: $r = 2(1 - \overline{P}_{0})$.

##### READv2

Likewise, for every pedigree replicate, the pseudo-haploid variant dataset produced by pileupCaller in Section Variant calling is provided to READv2 by BADGER, using the default parameters (–-norm_method median). Contrary to READ, the coefficient of relatedness *r* is directly obtained from the final output results of the software.

##### TKGWV2

BADGER applies TKGWV2 on every tested pair of post-processed alignment files, using the support 1000g-phase3 EUR population bed files and allele frequencies provided in [19] (1000GP3_22M_noFixed_noChr.bed and 1000GP3_EUR_1240K.frq, respectively). Here, note that providing TKGWV2 with a pre-defined dataset of allele frequencies incurs the risk of targeting positions that were not simulated by Ped-sim during a particular run, as BADGER makes successive use of pedigree simulations using a pre-processed 1000 g dataset, followed by the creation of consensus sequences (which will naturally exclusively contain reference alleles). Hence, to alleviate this potential source of reference bias, we first filter the provided 1000GP3_EUR_1240K.frq file by removing any position that was not found within the raw output VCF file previously emitted by Ped-sim. Also note that, contrary to all other candidate methods, TKGWV2 may only be applied on a single pair of individuals. Thus, applying this method on an entire pedigree replicate requires BADGER to employ a scatter-gather approach, by running the software on every tested pair of individuals, and subsequently merging the results.

### Statistical analysis and benchmark using badger.plots

Following the application of BADGER in multiple replicates, the statistical analysis and performance estimation of each method is handled using badger.plots : a command line interface, written as a companion software to BADGER. This software thus sequentially performs :

1. The deserialisation and consolidation of the results of each genetic relatedness estimation software, across all BADGER simulation replicates and sets of studied biological parameters.
2. The calculation of summary statistics regarding the classification performance of each method, for each biological parameter studied.
3. The estimation of the average accuracy and bias of each method’s *r*-coefficient calculation for each degree of relationship.
4. The generation of interactive plots summarising these performance statistics.

#### Estimation of classification performance

For every biological condition and method, we constructed confusion matrices confronting the predicted degrees of relationship for all pairwise comparisons, against the “true” degrees of relationship, defined by the topology of the template pedigree originally given as an input for BADGER. From these confusion matrices – one for every pedigree replicate – we calculated the Uniform Ordinal Classification Index (UOC) as a measure of a method’s overall classification performance [40]. Briefly, this performance metric, adapted from the ordinal classification index of Cardoso and Sousa [66], is bound between 0 and 1 (0 implying perfectly accurate classification), is insensitive to class-imbalance, and markedly takes into account the inherent relative order, and ranking distance separating two degrees of relationship. As such, the ordinal nature of estimating genetic relatedness is retained when estimating performance (e.g. misclassifying an “Unrelated” pairs of individuals as “First-degree” is more penalised than misclassifying them as “Second-degree”).

Here, our implementation of the UOC metric was incorporated into badger.plots by adapting the pseudo-code found in [66], and source code provided in [67]. The R source code which we wrote to reimplement this metric within badger.plots is encapsulated within a function signed cm_to_uoc. This function can be viewed on BADGER’s GitHub repository: https://github.com/MaelLefeuvre/BADGER/blob/v0.5.2/badger/src/badger-plots/badger.plots/R/get_performance_index.R#L181-L280. Note that the equation corresponding to our implementation directly corresponds to that found in [40] (Eq. 28), which is here shown for reference, and briefly described:

1 $$\begin{aligned} UOC_{\beta }^{\gamma } & = min \left\{ 1 - \frac{\sum \nolimits _{(r,c) \in path} (n_{r,c}/N_{r})1\!\!1_{\partial }(r)}{K' + \frac{K'}{K'^{\gamma }} \left( \sum \nolimits _{\forall (r,c)}(n_{r,c}/N_{r}) 1\!\!1_{\partial }(r) |r-c|^{\gamma } \right) ^{1/\gamma }} \right. \nonumber \\ & \qquad \qquad \left. + \frac{\beta }{K'} \sum \limits _{(r,c)\in path} \frac{n_{r,}}{N_{r}} 1\!\!1_{\partial }(r) |r - c|^{\gamma }\right\} \end{aligned}$$

Where *r* and *c* correspond to the row and column index of the confusion matrix, respectively. $n_{r,c}$ is the number of observations within a given cell. *N* is the total number of observations found within the confusion matrix. $1\!\!1_{\partial }(r)$ is an indicator function, specifying whether or not the considered row *r* contains any observations (in other terms, this value effectively equates to 1 if row *r* contains any observation, or 0 in the row contains none). $K'$ corresponds to the number of observed classes within the confusion matrix. Note here that $\beta$ and $\gamma$ are freely adjustable parameters; $\beta$ adjusts the penalisation factor for every deviation from the main diagonal, while $\gamma$ adjusts the magnitude of this penalty, based on the distance found between a given misclassification and the main diagonal. Calculating the $UOC_{\beta }^{\gamma }$ is therefore a minimisation task which is applied over the set of all consistent paths that can be traced along the confusion matrix, from cell (1, 1) to cell (K, K). Note that, as defined in [66] and [40], a path is considered “consistent” if and only if the relative order of the true classes for every pair of nodes comprising this path does not oppose the relative order of the predicted classes.

For the choice of the two arbitrary parameters $\beta$ and $\gamma$, we yet again follow the proposals of [40]: for $\gamma$, we simply implemented our calculation of the UOC metric using $\gamma = 1$; for $\beta$, we eliminate this parameter by integrating $UOC_{\beta }^{\gamma =1}$ along the range $\beta \in [0, 1]$

For every method and biological condition, the average UOC of every pedigree replicate is then calculated and plotted as a final aggregate summary statistic. 95% confidence intervals are directly estimated from the distribution of UOC values across all simulation replicates, using normal approximation, i.e.:

2 $$\begin{aligned} CI_{95\%}(UOC) = \mu \pm 1.96 \cdot \frac{\sigma }{\sqrt{n}} \end{aligned}$$

Where *n* corresponds to the number of obtained confusion matrices (one for every simulation replicate for the condition being studied), while $\mu$ and $\sigma$ respectively correspond to the average, and standard deviation of the *n* *UOC* values obtained for each simulation replicate. The value 1.96 approximates the z-score required to obtain a 2.5% area on each side of the standard normal distribution (normal approximation)

When applicable, estimates for the area under the curve (AUC) of UOC values of every method were obtained through trapezoidal integration, using the R package pracma version 2.4.4, and its associated function trapz [68].

#### Average accuracy and bias of relatedness coefficients

For every simulated degree of relationship, and across all tested methods and biological conditions, we calculate the average Root Mean Square Deviation (RMSD) and Mean Bias Error (MBE) between the *calculated* and the *expected* relatedness coefficients (*r*), in an effort to gain insight regarding the average accuracy and bias of each method. Note that, as many of the methods tested here do not *directly* compute *r*-coefficients, badger.plots must first derives this metric from the raw output of every method. Hence:

- As KIN estimates the Jacquard genetic identity coefficients of every pairwise relationship [69], we derived an *r*-coefficient from the provided $k_{1}$ and $k_{2}$ values, i.e.: $r = \frac{k_{1}}{2} + k_{2}$
- for READ and READv2, an *r*-coefficient can be calculated from the normalised $\overline{P_{0}}$ of a given pair, using the following formula: $r = 2\cdot (1-\overline{P_{0}})$
- for GRUPS-rs, an *r*-coefficient can be derived by first calculating normalised estimates of the $PWD_{i,j}^{obs}$ metric of a given pair *i*, *j*, which is obtained by dividing this raw estimate by the average expected distribution of unrelated pairs ($\widehat{PWD}_{i, j, unrelated}^{sim}$). It follows that the *r*-coefficient can be derived using the following equation: $r = 2(1 - \frac{PWD_{i,j}^{obs}}{\widehat{PWD}_{i,j,unrelated}^{sim}})$
- Finally, as both correctKin and TKGWV2 compute a kinship coefficient ($\phi$), the *r*-coefficient is simply obtained by multiplying this estimate by 2: $r = 2\cdot \phi$

Here, it must be noted that the distance separating the expected *r*-coefficient of a given degree of relationship from neighbouring distributions varies with the degree of relatedness, and is effectively halved for each additional degree separating two individuals. This implies that a given deviation from the expected average can have a greatly differing impact on the general accuracy, depending on the degree for which it is observed (e.g. a standard deviation of 0.1 for the *r*-coefficient between two individuals is insignificant when considering first-degree relationships, but would consistently cause misclassifications in the case of third-degree relationships).

Thus, to properly compare the accuracy and bias, both across a given method and degree of relatedness, we propose to first normalise the RMSD and MBE of a given relationship by the range of its theoretical distribution. This can be done by dividing the RMSD or MBE value by the distance separating the two midpoints found between the mean of a given relationship *k* and its neighbouring ones ($k-1$ and $k+1$).

3 $$\begin{aligned} nRMSD_{m,k} = \frac{\sqrt{\frac{\sum \nolimits _{i=1}^{i=N}(\widehat{r}_{k} - r_{m,k,i})^2}{N}}}{\frac{\widehat{r}_{k+1} - \widehat{r}_{k-1}}{2}} \end{aligned}$$

4 $$\begin{aligned} nMBE_{m,k} = \frac{\frac{\sum \nolimits _{i=1}^{i=N}(r_{m,k,i} - \widehat{r}_{k})}{N}}{\frac{\widehat{r}_{k+1} - \widehat{r}_{k-1}}{2}} \end{aligned}$$

where *m* and *k* represent a given method and degree of relatedness, respectively. $\widehat{r}_{k}$ is the expected relatedness coefficient of relationship *k*; $r_{m,k,i}$, the calculated relatedness coefficient for the $i^{th}$ pair of individuals, and *N*, the total amount of observations of a given relationship *k* ($r_{k}= \left\{0, 0.125, 0.25, 0.5, 1\right\}$). 95% confidence intervals for these metrics were calculated using the principles and methods described in [70, 71], i.e.:

5 $$\begin{aligned} nRMSD_{m,k} \in \left[ nRMSD_{m,k} \left( 1 - \sqrt{1 - \frac{1.96\sqrt{2}}{\sqrt{N-1}}}\right) ; RMSD_{m,k} \left( \sqrt{1 + \frac{1.96\sqrt{2}}{\sqrt{N - 1}}} - 1 \right) \right] \end{aligned}$$

6 $$\begin{aligned} nMBE_{m,k} \in \left[ nMBE_{m,k} \pm \frac{1.96 \cdot \sigma _{m,k}}{\sqrt{N}}\right] \end{aligned}$$

where $\sigma _{m,k}$ is the population standard deviation of the relatedness coefficients obtained from method *m*, and belonging to relationship *k*.

## Supplementary Information

Additional file 1: Supplementary Tables. 1. Supplementary Table S1: Denominator values used for the normalisation of RMSD and MBE estimates. 2. Supplementary Table S2: Description of the pairwise relationships contained within the input pedigrees used throughout this study. 3. Supplementary Table S3: Nominal multiclass performance statistics, obtained across every parameter configuration, tested method, and expected degree of relatedness 4. Supplementary Table S4: Average pairwise SNP overlap reported by the correctKin, GRUPS-rs, TKGWV2 and READv2 methods, across the benchmark parameter space. 5. Supplementary Table S5: Comparing the sensitivity of outbred relationships vs. non-intermediate relationships involving inbred individuals.

Additional file 2: Supplementary materials. 1. Supplementary Figures S1-S20. 2. Description of the pmd-mask command line utility. 3. List of key parameters. 4. Key Resources Table.

## Data Availability

The main software BADGER and its companion program badger.plots are available on Github under the GPLv3 license [72]. A snapshot of the version used during this study is available at Zenodo [73]. Source code, documentation, and precompiled binaries for the in-house pmd-mask utility software are publicly available on Github under the GPLv3 license [74]. A snapshot of the version used during this study is available at Zenodo [75]. The raw output of every tested genetic relatedness estimation method generated throughout this benchmark, and BADGER configuration files required to fully reproduce the results of this study are available at Zenodo [76]. Each and every .yaml configuration file (containing all key parameters for the BADGER simulation pipeline) used to generate the simulations of this benchmark, are available in this Zenodo archive, and can be used to fully replicate the results presented here. Both BADGER and the benchmark presented in this manuscript rely on several publicly available datasets, namely: – The 1000 genomes phase3 dataset version v20130502 [32] – The Allen Ancient DNA Resource version v52.2 [60] – The HapMap phase II genetic recombination map, version v2011-01-b37 [65] – The refined sex-specific genetic recombination map from (Bhérer et al. 2017) [53] – The human crossover interference parameters from (Caballero et al. 2019) [36] – Supporting files and datasets from the software TKGWV2 [19] A comprehensive list of all software and publicly archived datasets analysed for the purposes of this study is available as a key resource table in the supplementary materials (Additional file 2: Section “Key Resources Table”).
