# Supplementary material for: Evaluating the performance of ancient DNA genetic relatedness estimation methods using high-fidelity pedigree simulations
Source: Genome Biol. 2026 Mar 9;27:132. doi: 10.1186/s13059-026-04016-y (PMC13081257; doi:10.1186/s13059-026-04016-y)
Supplement: Supplementary file 2 — Additional file 2: Supplementary materials. 1. Supplementary Figures S1-S20. 2. Description of the pmd-mask command line utility. 3. List of key parameters. 4. Key Resources Table. [file 13059_2026_4016_MOESM2_ESM.pdf]

Supplementary Materials:

Evaluating the performance of ancient DNA genetic relatedness estimation methods using high-fidelity pedigree simulations.

Contents

1 Supplementary Figures **4**

1.1 Figure S1: Alternate template pedigree including inbred individuals . . 4

1.2 Figure S2: Alternative input pedigrees used to evaluate the impact of inbreeding on the normalisation procedure of correctKin, KIN and READv2 5

1.3 Figure S3: Comparing the performance of READv2 against its predecessor, READ 6

1.4 Figure S4: Accuracy and bias of r-coefficients as a function of sequencing depth (pmd-mask) 7

1.5 Figure S5: Accuracy and bias of r-coefficients as a function of sequencing depth (mapDamage2) 8

1.6 Figure S6: Accuracy and bias of r-coefficients as a function of sequencing depth (trimBam) 9

1.7 Figure S7: Full grid of confusion matrices and UOC values across increasing values of sequencing depth (mapDamage2) 10

1.8 Figure S8: Full grid of confusion matrices and UOC values across increasing values of sequencing depth (trimBam) 11

1.9 Figure S9: Accuracy and bias of r-coefficients as a function of contamination rate (AFR) 12

1.10 Figure S10: Accuracy and bias of r-coefficients as a function of contamination (GBR) 13

1.11 Figure S11: Full grid of confusion matrices and UOC values across increasing values of contamination (AFR) 14

1.12 Figure S12: Full grid of confusion matrices and UOC values across increasing values of contamination (GBR) 15

1.13 Figure S13: Ancestry proportions of admixed American populations . 16

1.14 Figure S14: Average heterozygosity rate of the European CEU population, and admixed American populations 17
1.15 Figure S15: Impact of admixture on the accuracy and bias of r-coefficients 18
1.16 Figure S16: Confusion matrices and UOC values across increasing values of sequencing depth (ASW) 19
1.17 Figure S17: Accuracy and bias of r-coefficients estimates for pairwise comparisons involving inbred individuals 20
1.18 Figure S18: Impact of inbreeding on the accuracy and bias of r-coefficients estimates for pairwise comparisons involving outbred individuals 21
1.19 Figure S19: Directed acyclic graph describing BADGER's simulation pipeline 22
1.20 Figure S20: Input pedigree definition file of GRUPS-rs 23

2 Description of the pmd-mask command line utility 24

2.1 Rationale, behaviour and workflow description 24
2.2 Pseudo-code describing the main algorithm of pmd-mask 27

3 List of key parameters 28

4 Key Resources Table 31

1 Supplementary Figures

1.1 Figure S1: Alternate template pedigree including inbred individuals

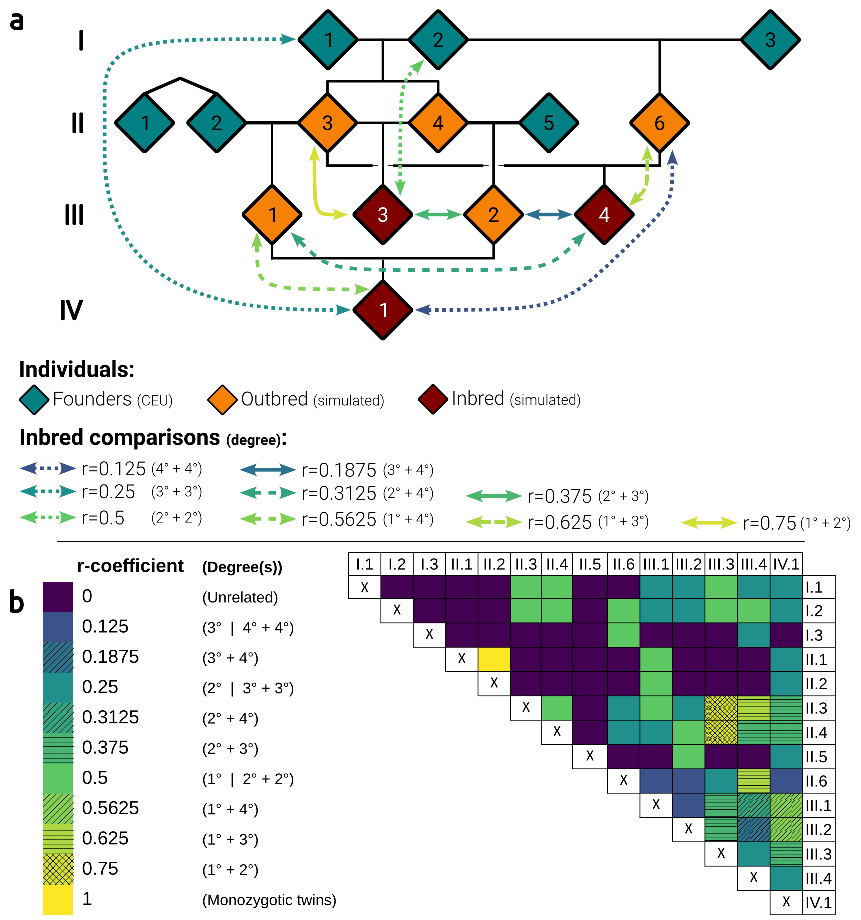

Fig. S1: Alternate template pedigree including three inbred individuals. **a**: Diagram of the alternative template pedigree, provided to BADGER when evaluating the impact of close inbreeding. Coloured arrows denote the pairwise relationships investigated during the inbreeding benchmark. Additional inbred individuals are coloured in dark red, with individuals III.3, III.4 and IV.1 being the result of a mating respectively involving two siblings, two half-siblings, and two first-cousins. **b**: Pairwise matrix of the relationships defined by the simulated pedigree shown in (a). A complete description of the pairwise relationships contained within the input pedigrees used throughout this study is described in Supplementary Table S2.

1.2 Figure S2: Alternative input pedigrees used to evaluate the impact of inbreeding on the normalisation procedure of correctKin, KIN and READv2

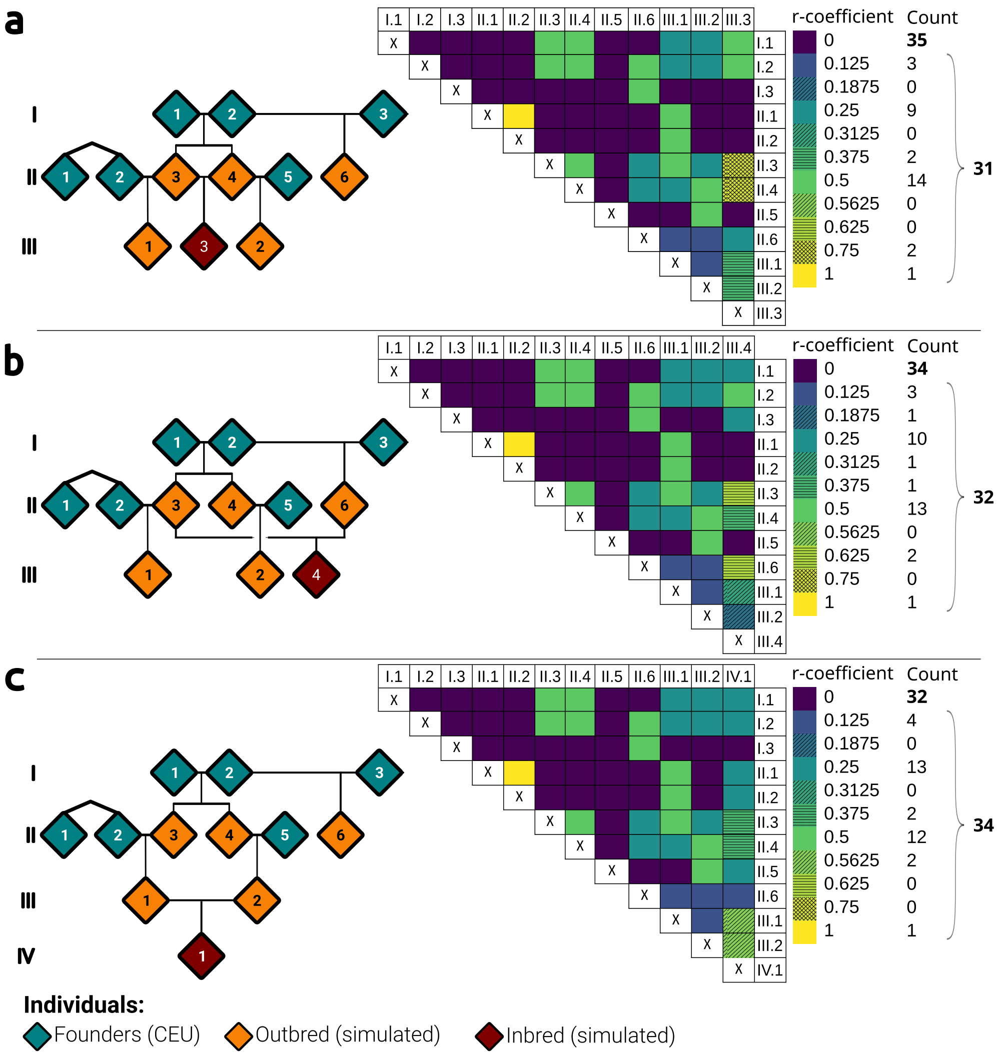

Fig. S2: Diagram and pairwise relationship matrix of three alternative pedigree topologies, given as input to correctKin, KIN and READv2 methods, where only one out of the three inbred individuals (III.3, III.4 and IV.1) is included in the tested cohort. **a**: Full-siblings scenario. **b**: Half-siblings scenario. **c**. First-cousins scenario.

1.3 Figure S3: Comparing the performance of READv2 against its predecessor, READ

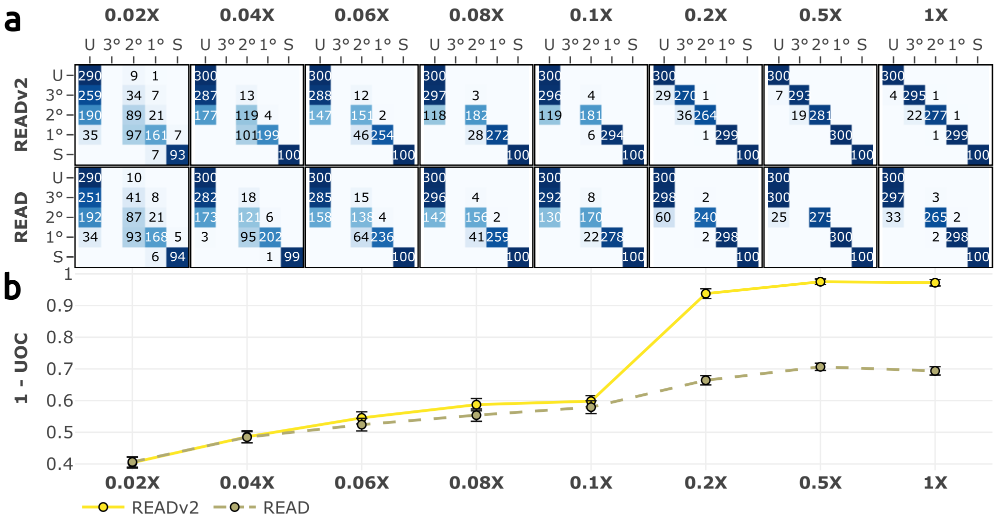

Fig. S3: Benchmark results across increasing values of sequencing depth for the READ (dashed tan line) method and its updated version, READv2 (solid yellow line). **a**: Confusion matrices of the READ and READv2 methods, confronting expected and predicted relationships. Expected and predicted values are displayed in rows and columns, respectively. 1°, 2°, 3° correspond to first-, second-, and third-degree relationships, respectively, U corresponds to "unrelated individuals", and S to "self" (monozygotic twins). **b**: UOC values summarizing the classification performance of each method for the considered sequencing depths. Higher values of $1 - UOC$ indicate higher performance. Error bars represent 95% confidence intervals around the mean UOC values.

1.4 Figure S4: Accuracy and bias of r-coefficients as a function of sequencing depth (pmd-mask)

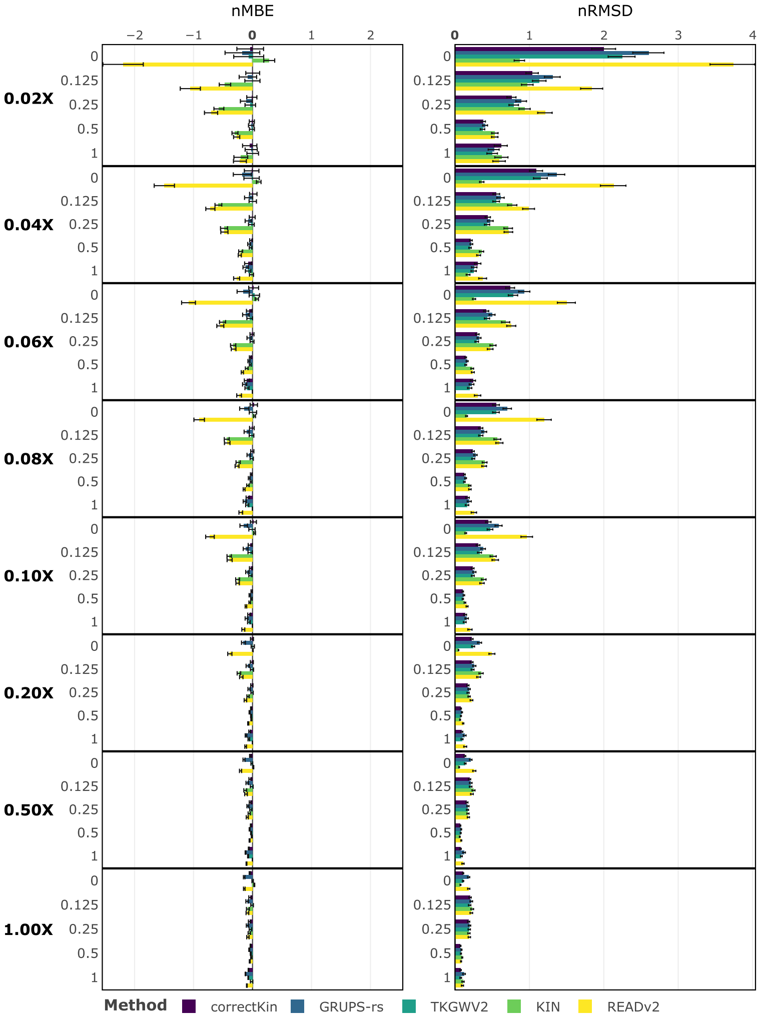

Fig. S4: Normalized estimates of MBE (left column) and $RMSD$ (right column), across all evaluated methods (bar colours), sequencing depths (rows), and expected relatedness coefficients (y-axis ticks), using sample alignment files processed through `pmd-mask`. Increasing values of $nRMSD$ indicate lower accuracy when estimating relatedness coefficients. $nMBE$ values that deviate furthest from zero indicate higher bias, with positive and negative values highlighting a tendency towards over- or under-estimating r-coefficients, respectively. Error bars represent $CI_{95\%}$ for the given estimate.

1.5 Figure S5: Accuracy and bias of r-coefficients as a function of sequencing depth (mapDamage2)

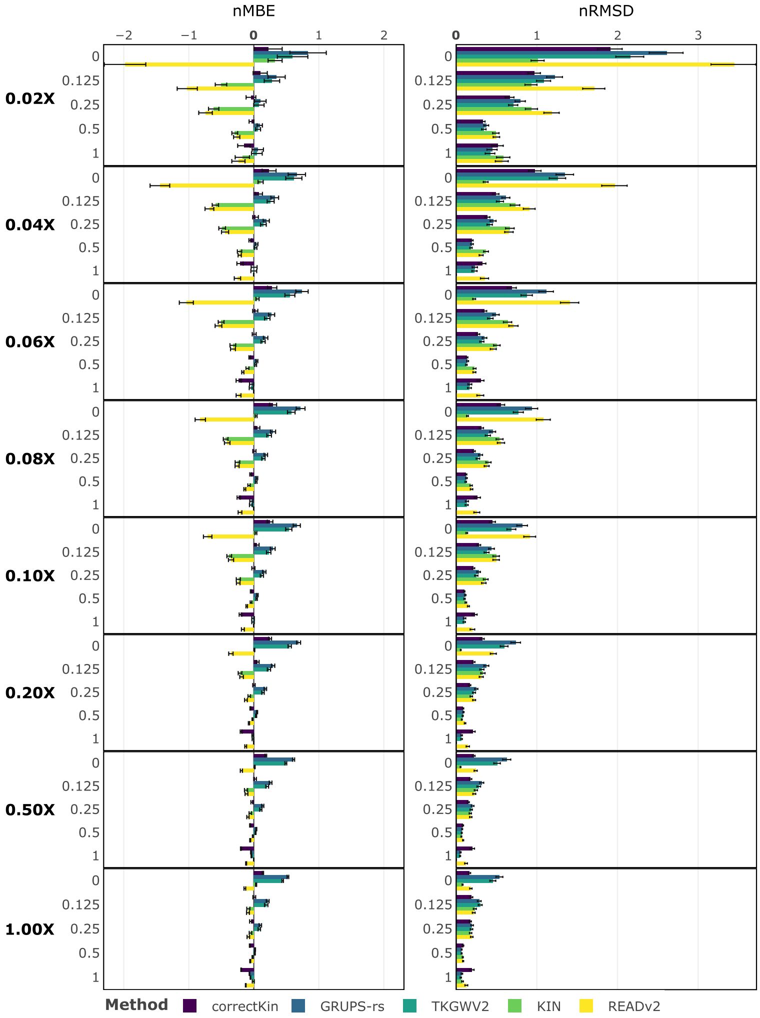

Fig. S5: *nMBE* (left column) and *nRMSD* (right column) estimates, across all evaluated methods (bar colours), sequencing depths (rows), and expected relatedness coefficients (y-axis ticks), using sample alignment files processed through `mapDamage2`. Increasing values of *nRMSD* indicate lower accuracy when estimating relatedness coefficients. *nMBE* values that deviate furthest from zero indicate higher bias, with positive and negative values highlighting a tendency towards over- or under-estimating r-coefficients, respectively. Error bars represent $CI_{95\%}$ for the given estimate.

1.6 Figure S6: Accuracy and bias of r-coefficients as a function of sequencing depth (trimBam)

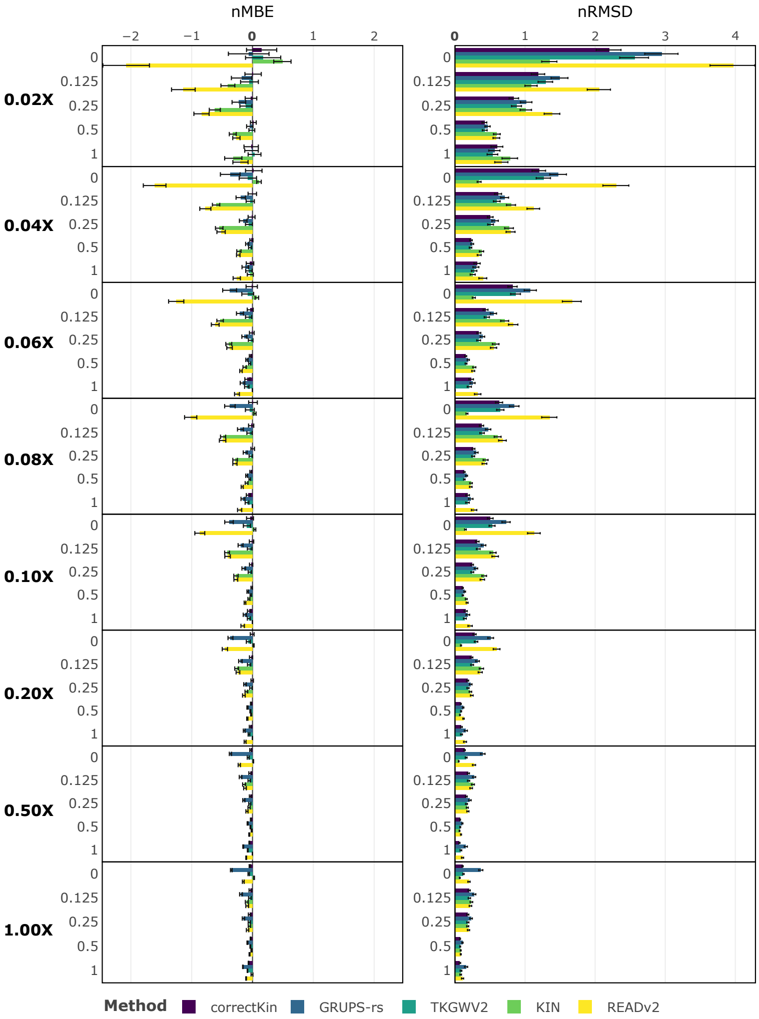

Fig. S6: $nMBE$ (left column) and $nRMSD$ (right column) estimates, across all evaluated methods (bar colours), sequencing depths (rows), and expected relatedness coefficients (y-axis ticks), using sample alignment files processed through `trimBam`. Increasing values of $nRMSD$ indicate lower accuracy when estimating relatedness coefficients. $nMBE$ values that deviate furthest from zero indicate higher bias, with positive and negative values highlighting a tendency towards over- or under-estimating r-coefficients, respectively. Error bars represent $CI_{95\%}$ for the given estimate.

1.7 Figure S7: Full grid of confusion matrices and UOC values across increasing values of sequencing depth (mapDamage2)

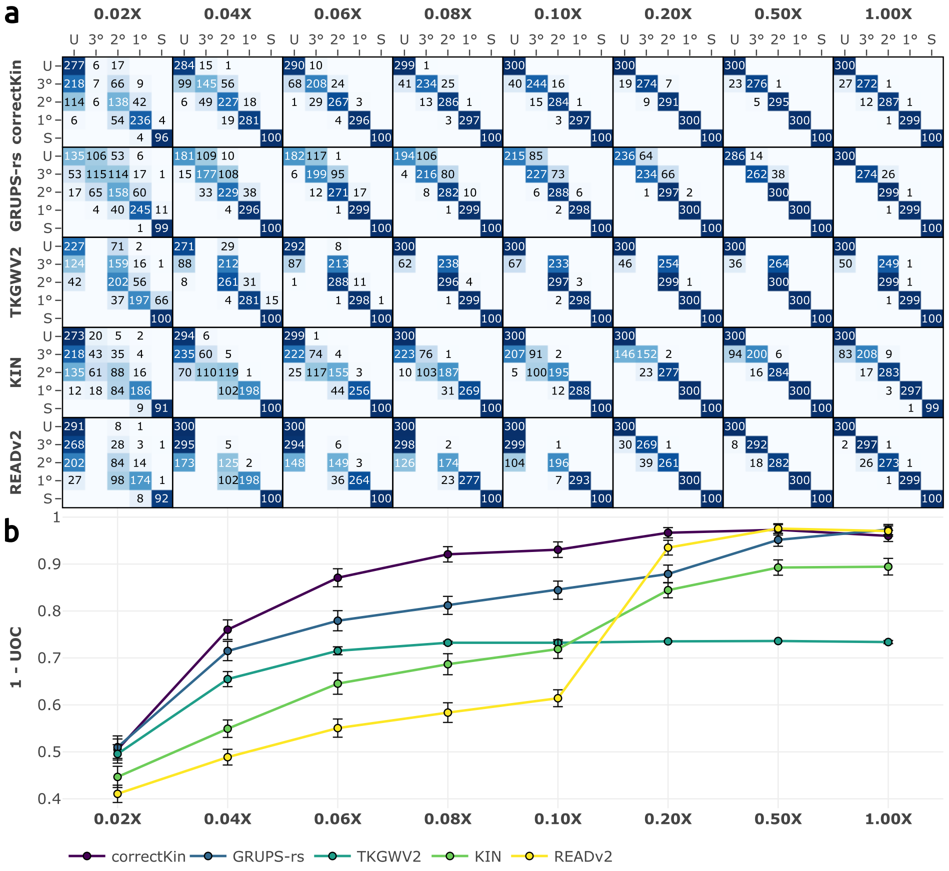

Fig. S7: Benchmark results across increasing values of sequencing depth, using sample alignment files processed through mapDamage2 *post-mortem* damage rescaling software. **a**: Confusion matrices of the five tested methods confronting expected and predicted relationships. Expected and predicted values are displayed in rows and columns, respectively. 1°, 2°, 3° correspond to first-, second-, and third-degree relationships, respectively, U corresponds to "unrelated individuals", and S to "self" (monozygotic twins). **b**: UOC values summarizing the classification performance of each method for the considered sequencing depths. Higher values of $1 - UOC$ indicate higher performance. Error bars represent 95% confidence intervals around the mean UOC values.

1.8 Figure S8: Full grid of confusion matrices and UOC values across increasing values of sequencing depth (trimBam)

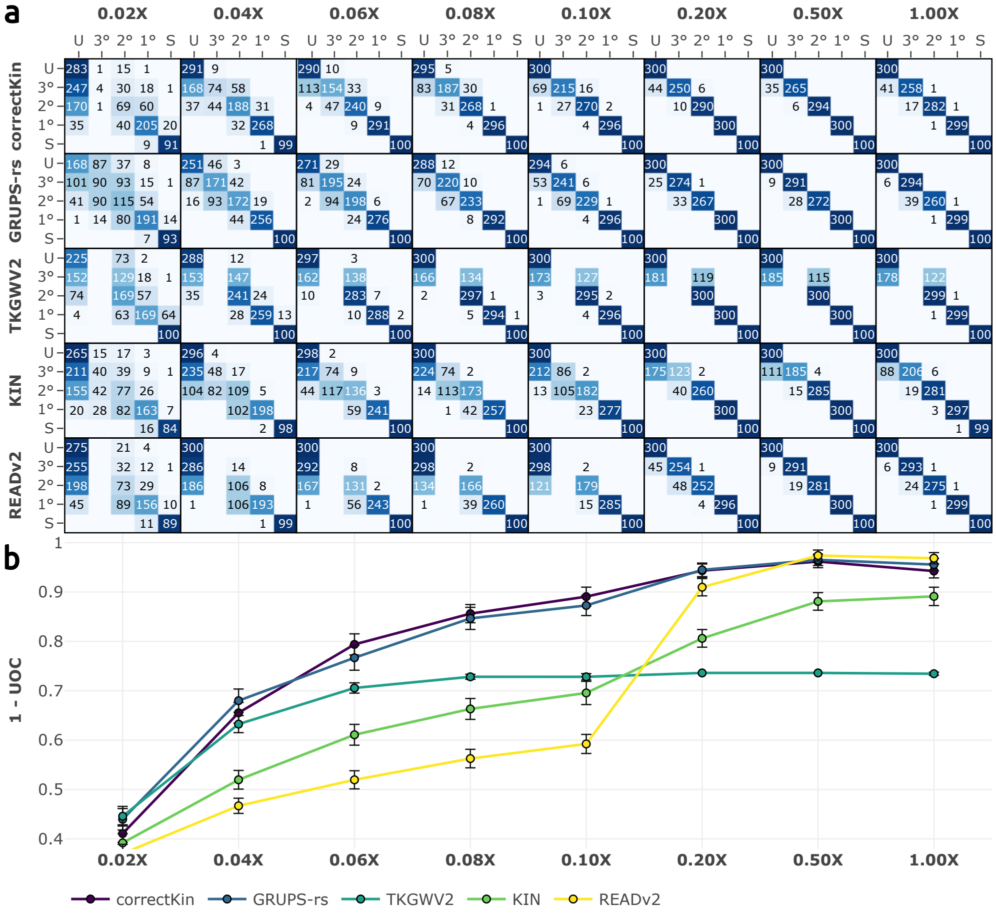

Fig. S8: Benchmark results across increasing values of sequencing depth, using sample alignment files processed through trimBam read-end trimming software. **a**: Confusion matrices of the five tested methods confronting expected and predicted relationships. Expected and predicted values are displayed in rows and columns, respectively. 1°, 2°, 3° correspond to first-, second-, and third-degree relationships, respectively, U corresponds to "unrelated individuals", and S to "self" (monozygotic twins). **b**: UOC values summarizing the classification performance of each method for the considered sequencing depths. Higher values of $1 - UOC$ indicate higher performance. Error bars represent 95% confidence intervals around the mean UOC values.

1.9 Figure S9: Accuracy and bias of r-coefficients as a function of contamination rate (AFR)

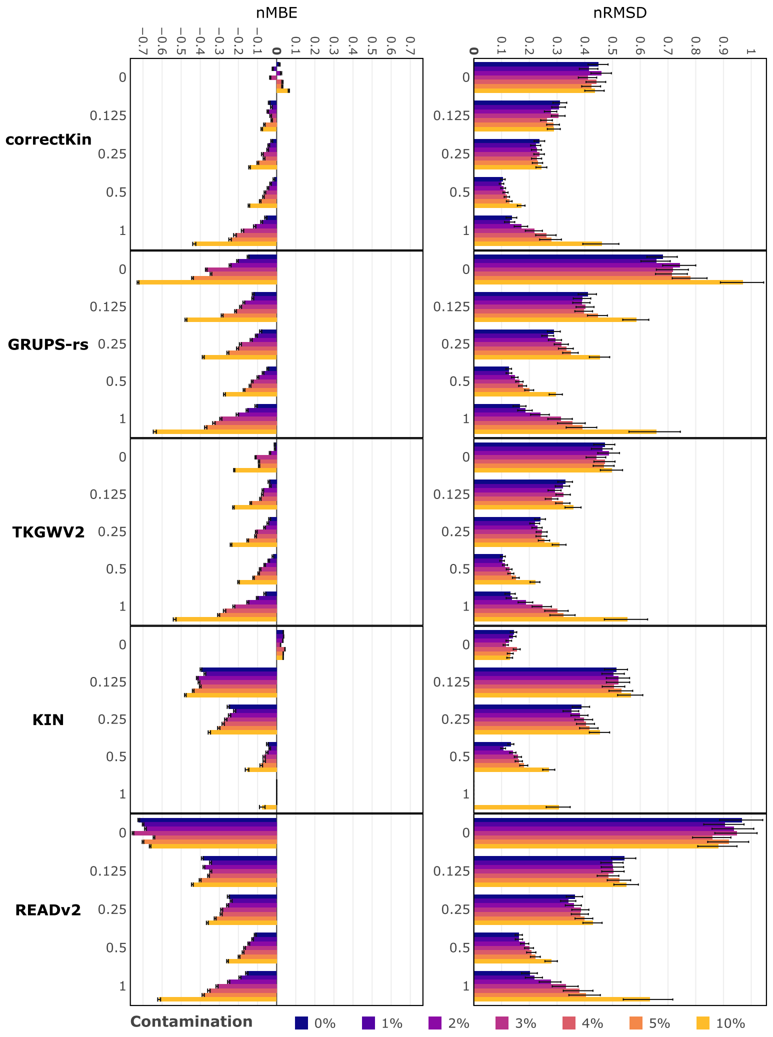

Fig. S9: $nMBE$ (left column) and $nRMSD$ (right column) estimates, across all evaluated methods (rows), rates of contamination from an AFR individual (bar colours) [32], and expected relatedness coefficients (y-axis ticks). Increasing values of $nRMSD$ indicate lower accuracy when estimating relatedness coefficients. $nMBE$ values that deviate furthest from zero indicate higher bias, with positive and negative values highlighting a tendency towards over- or under-estimating r-coefficients, respectively. Error bars represent $CI_{95\%}$ for the given estimate.

1.10 Figure S10: Accuracy and bias of r-coefficients as a function of contamination (GBR)

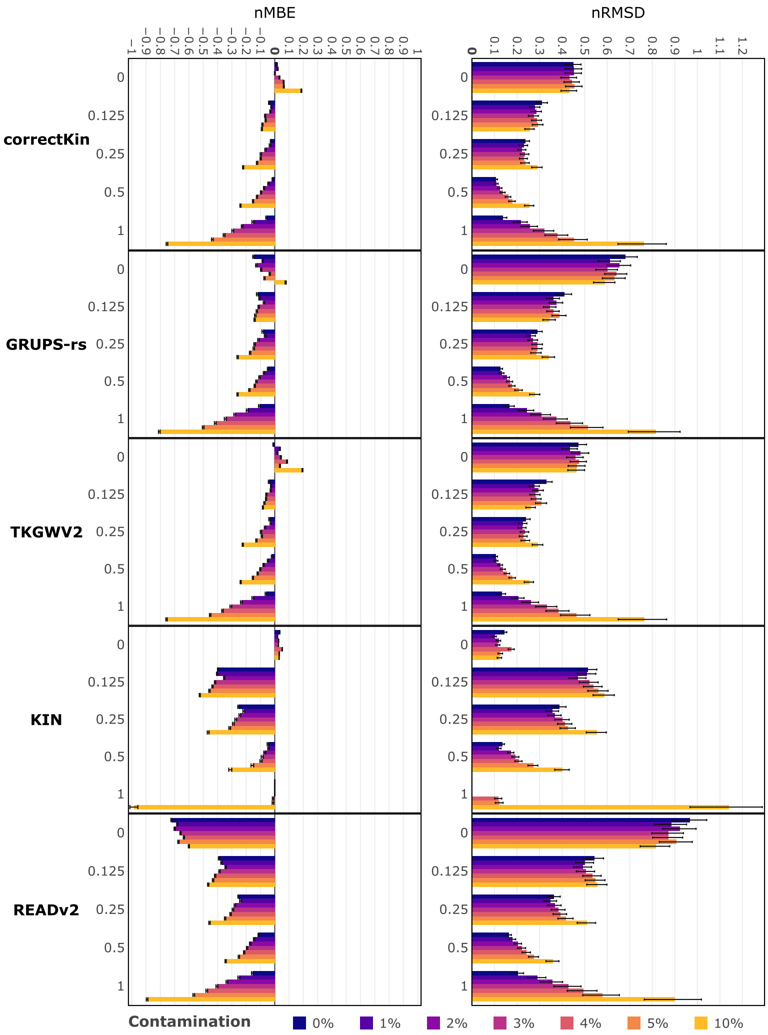

Fig. S10: $nMBE$ (left column) and $nRMSD$ (right column) estimates, across all evaluated methods (rows), rates of contamination from a GBR individual (bar colours)[32], and expected relatedness coefficients (y-axis ticks). Increasing values of $nRMSD$ indicate lower accuracy when estimating relatedness coefficients. $nMBE$ values that deviate furthest from zero indicate higher bias, with positive and negative values highlighting a tendency towards over- or under-estimating r-coefficients, respectively. Error bars represent $CI_{95\%}$ for the given estimate.

1.11 Figure S11: Full grid of confusion matrices and UOC values across increasing values of contamination (AFR)

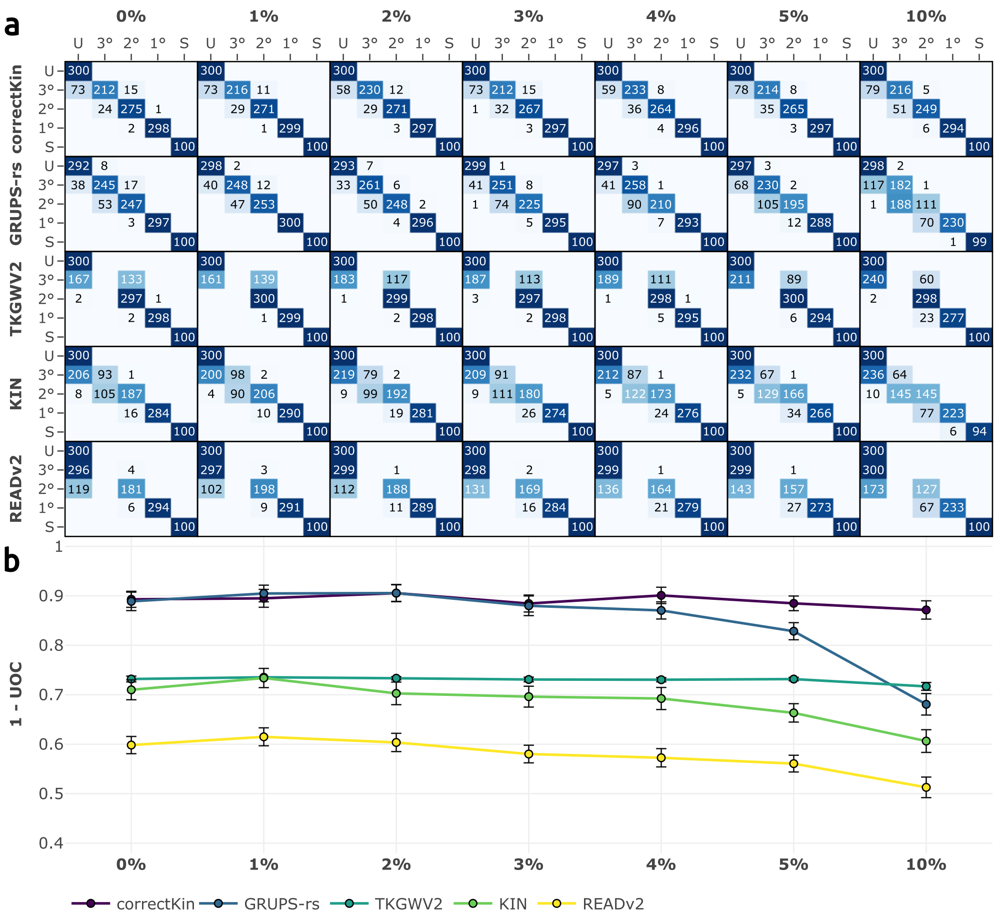

Fig. S11: Benchmark results across increasing values of modern human contamination, using the AFR population [32] as a source of contaminating individuals. **a**: Confusion matrices of the five tested methods, confronting expected and predicted relationships. Expected and predicted values are displayed in rows and columns, respectively. 1°, 2°, 3° correspond to first-, second-, and third-degree relationships, respectively, U corresponds to "unrelated individuals", and S to "self" (monozygotic twins). **b**: UOC values summarizing the classification performance of each method for the considered contamination rate. Higher values of $1 - UOC$ indicate higher performance. Error bars represent 95% confidence intervals around the mean UOC values.

1.12 Figure S12: Full grid of confusion matrices and UOC values across increasing values of contamination (GBR)

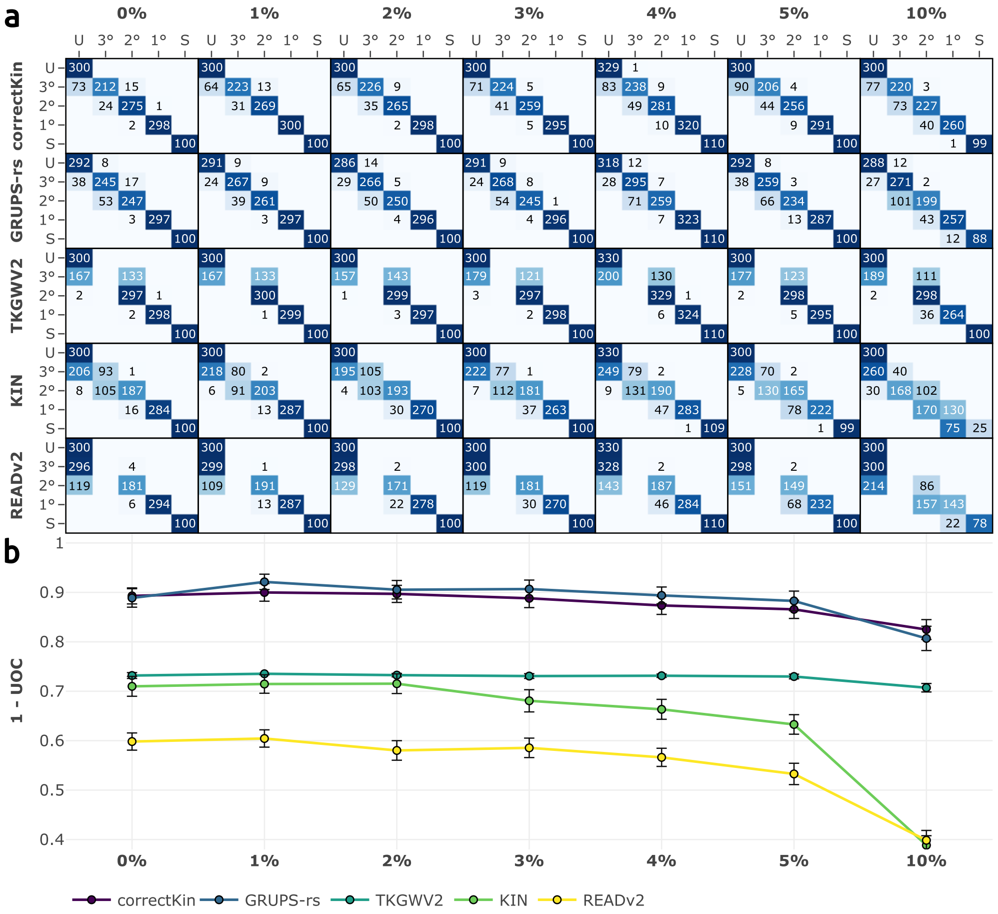

Fig. S12: Benchmark results across increasing values of modern human contamination, using the *GBR* population [32] as a source of contaminating individuals. **a**: Confusion matrices of the five tested methods, confronting expected and predicted relationships. Expected and predicted values are displayed in rows and columns, respectively. 1°, 2°, 3° correspond to first-, second-, and third-degree relationships, respectively, U corresponds to "unrelated individuals", and S to "self" (monozygotic twins). **b**: UOC values summarizing the classification performance of each method for the considered contamination rate. Higher values of $1 - UOC$ indicate higher performance. Error bars represent 95% confidence intervals around the mean UOC values.

1.13 Figure S13: Ancestry proportions of admixed American populations

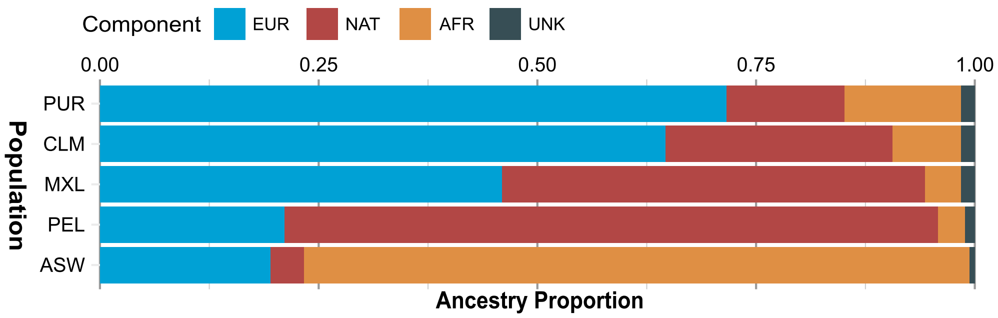

Fig. S13: Ancestry proportions of admixed American populations used during this study. The proportions of this plot were generated using the local ancestry inference results of (Martin et al. 2017) [47] (https://personal.broadinstitute.org/armartin/tgp_admixture). **AFR**: African; **EUR**: European; **NAT**: Native American; **UNK**: Unknown

1.14 Figure S14: Average heterozygosity rate of the European CEU population, and admixed American populations

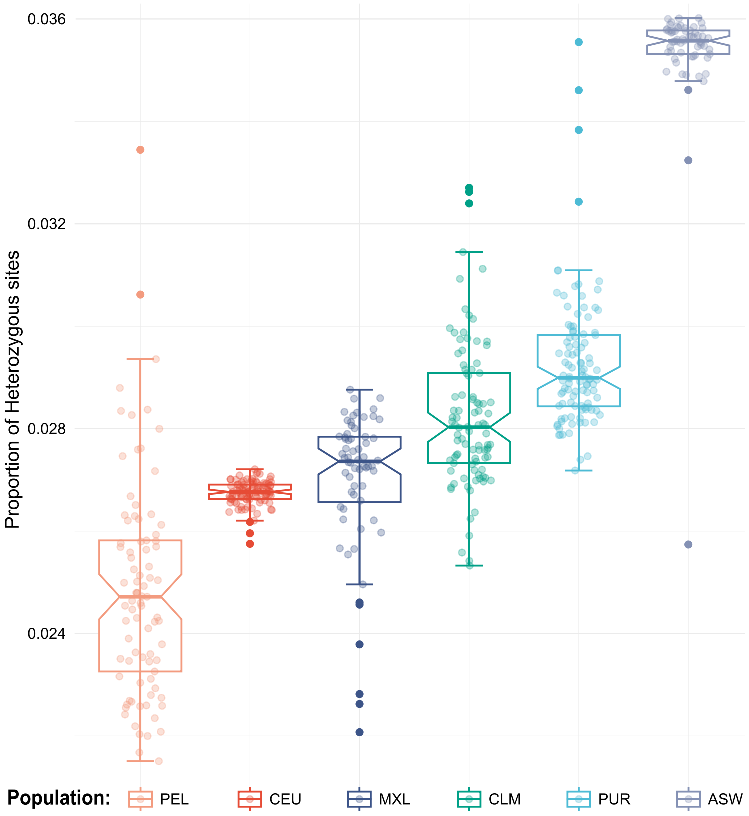

Fig. S14: Sample-wise distribution of the proportions of heterozygous sites of every 1000g-phase3 sample, according to their assigned population. Per-sample counts and proportions of heterozygous sites were directly calculated from the base dataset of the 1000g-phase3 project, using `bcftools stats` [52]. Note that these counts only take SNPs into account. Boxplot notches represent the 95% confidence interval of the median. Whiskers of each boxplot extend from the maximum to the minimum value found within the range $[Q_1 - 1.5 \cdot IQR; Q_3 + 1.5 \cdot IQR]$.

1.15 Figure S15: Impact of admixture on the accuracy and bias of r-coefficients

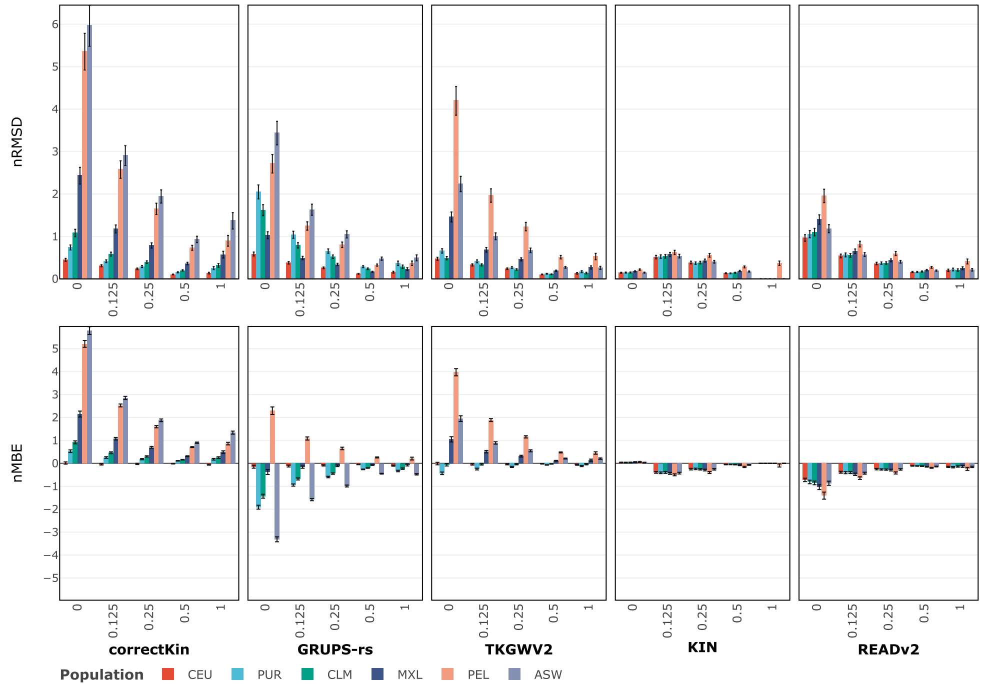

Fig. S15: $nRMSD$ (top row) and $nMBE$ (bottom row) estimates, across all evaluated methods (columns), source populations (bar colours), and expected relatedness coefficients (x-axis ticks), at a simulated sequencing depth of 0.1X. Increasing values of $nRMSD$ indicate lower accuracy when estimating relatedness coefficients. $nMBE$ values that deviate furthest from zero indicate higher bias, with positive and negative values highlighting a tendency towards over- or under-estimating r-coefficients, respectively. Error bars represent $CI_{95\%}$ for the given estimate.

1.16 Figure S16: Confusion matrices and UOC values across increasing values of sequencing depth (ASW)

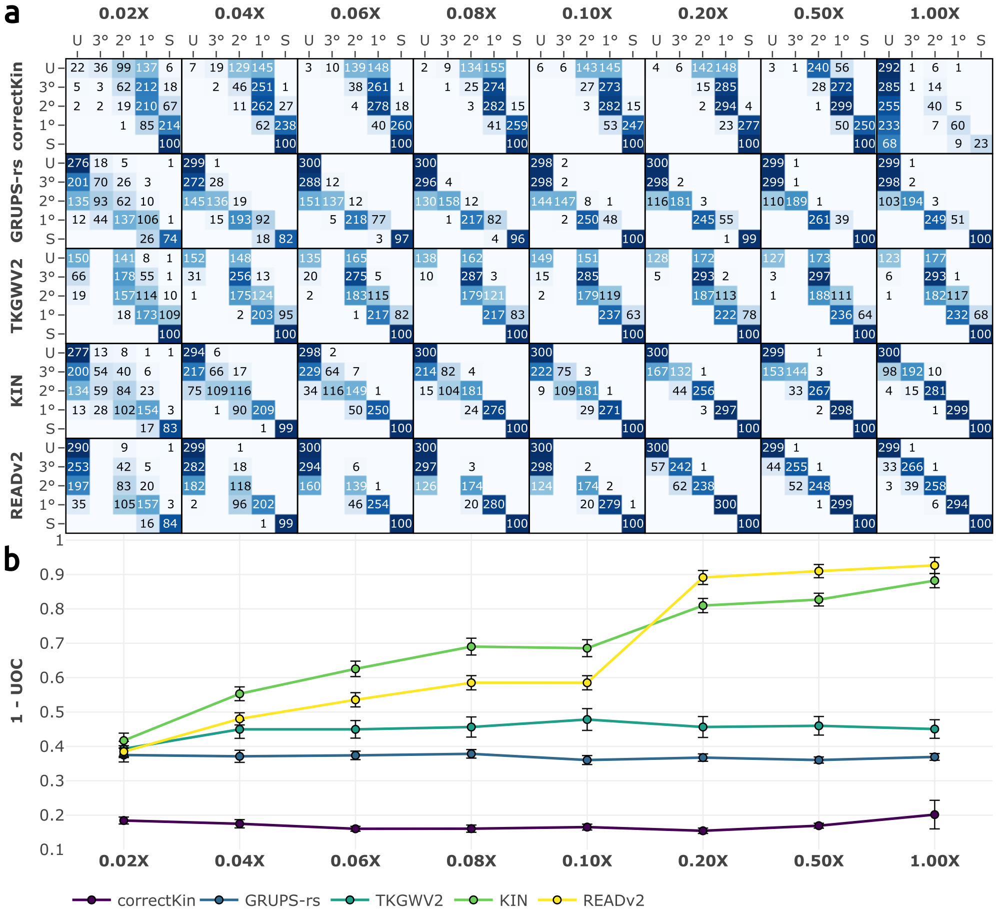

Fig. S16: Benchmark results across increasing values of sequencing depth, using admixed *ASW* individuals as a source population for pedigree individuals [32]. **a**: Confusion matrices of the five tested methods confronting expected and predicted relationships. Expected and predicted values are displayed in rows and columns, respectively. 1°, 2°, 3° correspond to first-, second-, and third-degree relationships, respectively, *U* corresponds to "unrelated individuals", and *S* to "self" (monozygotic twins). **b**: UOC values summarizing the classification performance of each method for the considered sequencing depths. Higher values of $1 - UOC$ indicate higher performance. Error bars represent 95% confidence intervals around the mean UOC values.

1.17 Figure S17: Accuracy and bias of r-coefficients estimates for pairwise comparisons involving inbred individuals

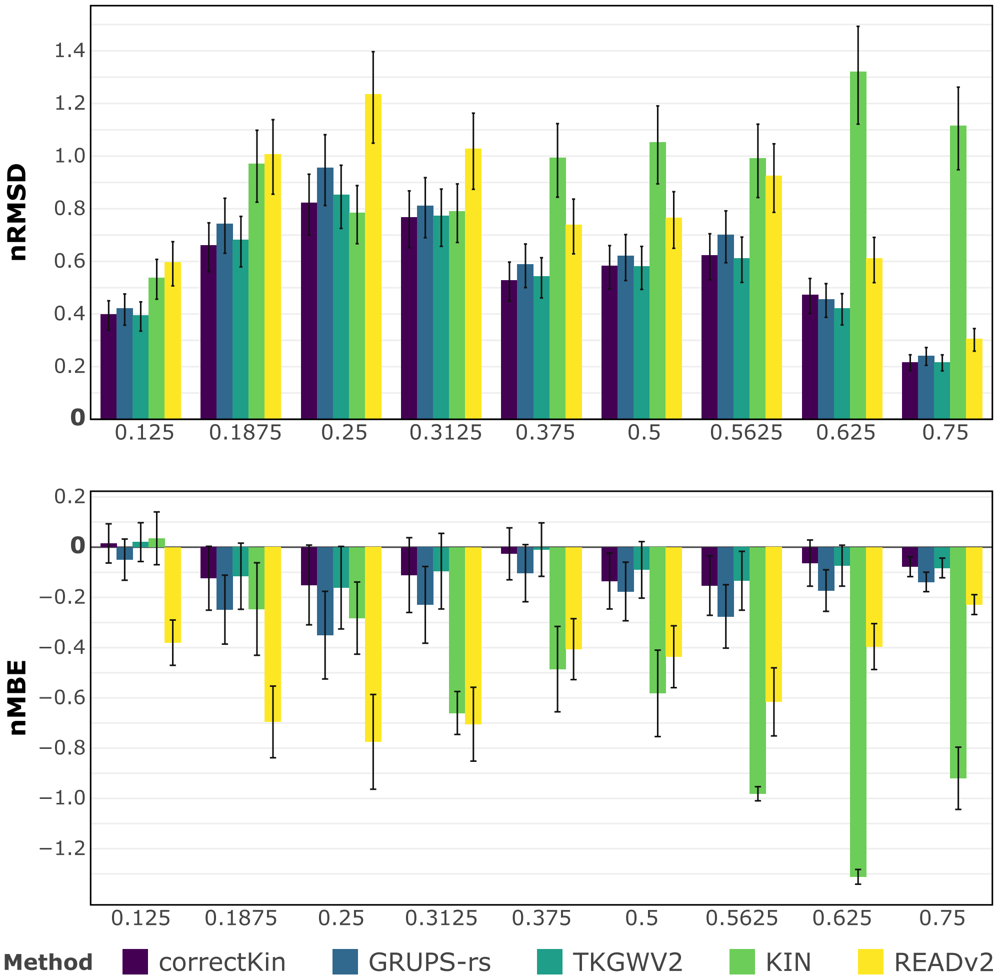

Fig. S17: $nRMSD$ (top row) and $nMBE$ (bottom column) estimates, obtained when simulating inbreeding, across all evaluated methods (bar colours) and expected relatedness coefficients (x-axis ticks). Increasing values of $nRMSD$ indicate lower accuracy when estimating relatedness coefficients. $nMBE$ values that deviate furthest from zero indicate higher bias, with positive and negative values highlighting a tendency towards over- or under-estimating r-coefficients, respectively. Error bars represent $CI_{95\%}$ for the given estimate.

1.18 Figure S18: Impact of inbreeding on the accuracy and bias of r-coefficients estimates for pairwise comparisons involving outbred individuals

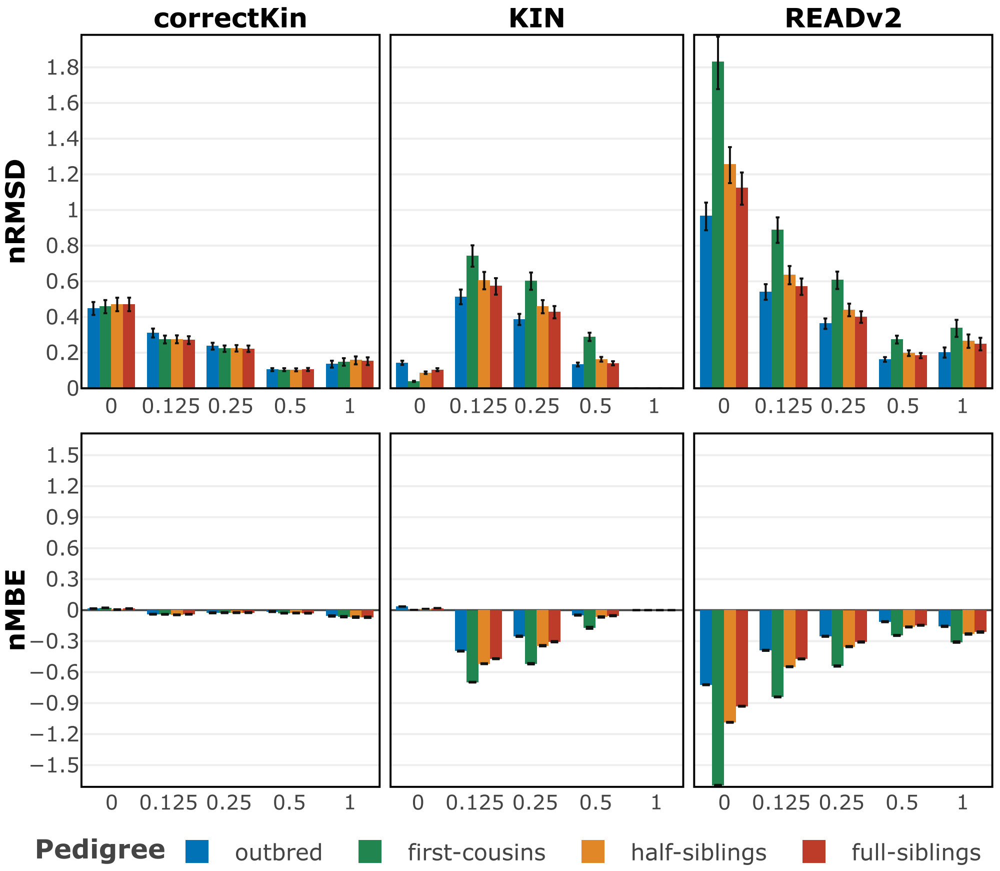

Fig. S18: $nRMSD$ (top row) and $nMBE$ (bottom column) estimates, obtained when simulating inbreeding, across all evaluated methods (columns), expected relatedness coefficients (x-axis ticks) and pedigree scenarii (bar colours). Increasing values of $nRMSD$ indicate lower accuracy when estimating relatedness coefficients. $nMBE$ values that deviate furthest from zero indicate higher bias, with positive and negative values highlighting a tendency towards over- or under-estimating r-coefficients, respectively. Error bars represent $CI_{95\%}$ for the given estimate.

1.19 Figure S19: Directed acyclic graph describing BADGER's simulation pipeline

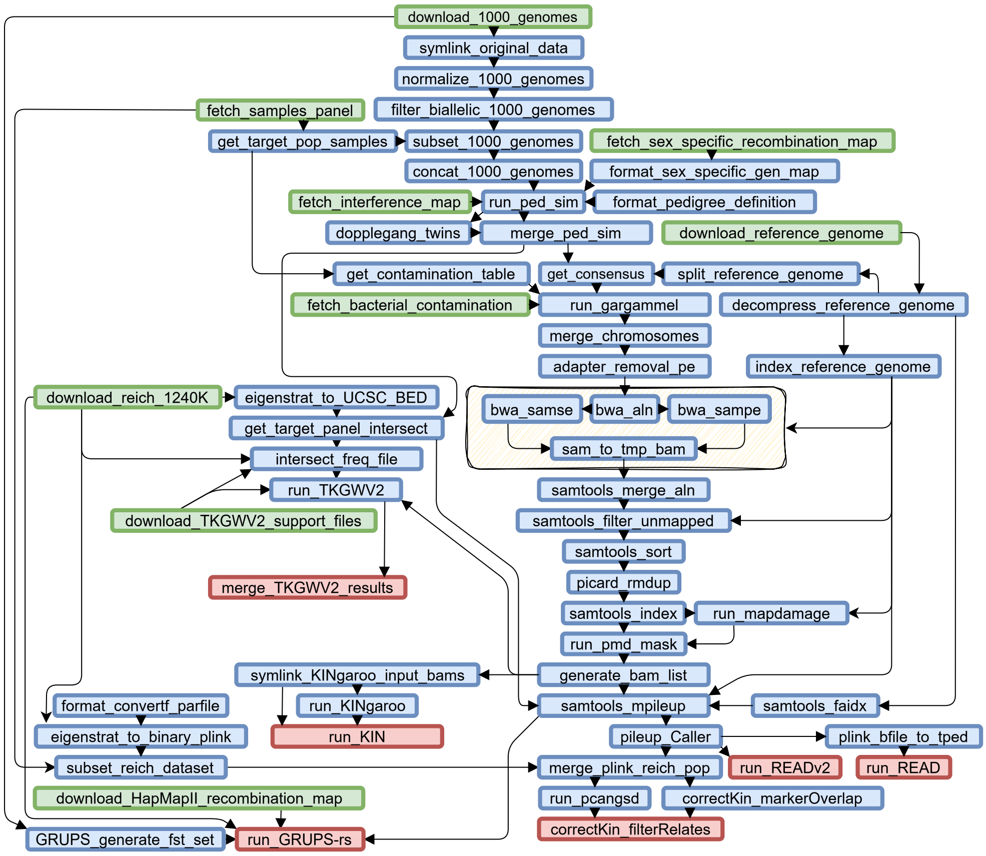

Fig. S19: Complete directed acyclic graph of the BADGER workflow, given the input and parameters provided throughout this study. Node names follow those of the snakemake rules found in BADGER's source code. Green nodes represent data entry points which are automatically downloaded by BADGER. Red nodes represent snakemake rules that are targeted by BADGER by default. The yellow cluster denotes a grouped data input for the reference genome.

1.20 Figure S20: Input pedigree definition file of GRUPS-rs

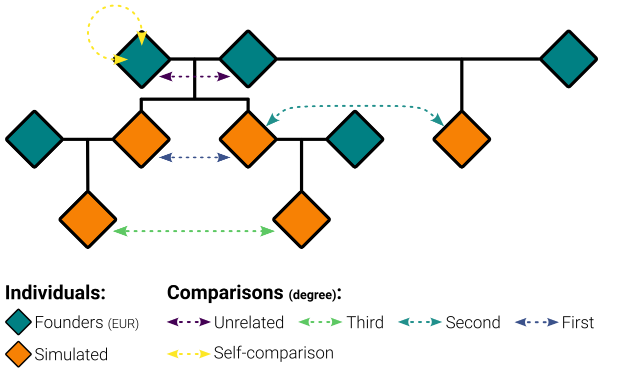

Fig. S20: Diagram of the input template pedigree definition file provided to the GRUPS-rs method throughout this study.

2 Description of the pmd-mask command line utility

2.1 Rationale, behaviour and workflow description

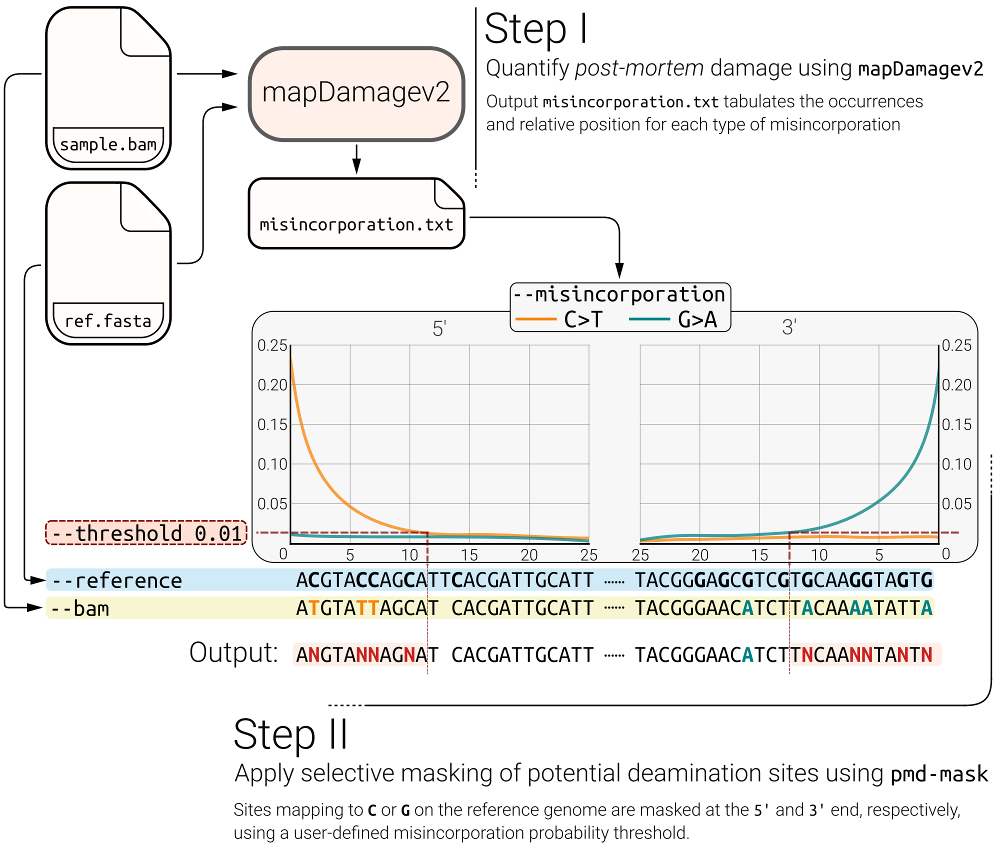

Fig. S21: Summary diagram describing the main process, required inputs, and workflow surrounding the `pmd-mask` command-line utility.

`pmd-mask` is a simple command-line pre-processing tool written in the Rust programming language, which masks the positions from reads that are likely to be impacted by *post-mortem* damage (PMD). Briefly, `pmd-mask` leverages nucleotide– and position-specific misincorporation rate estimates emitted from the mapDamage2 software (typically, in the form of a `misincorporation.txt` output file) to apply soft-trimming on the read extremities, until the PMD rate reaches a user defined threshold

by setting candidate nucleotides to ‘N’ and their base-quality to 0. Note that the default threshold is here defined as a misincorporation rate of 0.01, but may be modified at leisure by the user, using the `--threshold` argument. Note that, in its current state, the `pmd-mask` algorithm only considers the misincorporation rate found at a given position, and the genotype found in the *reference* genome. Therefore, nucleotides at the extremities of a read are masked, regardless of the actual genotype observed at a given position (Supplementary Figure S21). Pseudo-code snippets, summarizing the main loop of the program can be read in Algorithm-1. Here, the devised approach is one that i) is expected to incur less potential bias than when applying PMD-rescaling through mapDamage2 (i.e. using its provided `--rescale` flag), and ii) carries the benefit of mitigating the loss of information usually displayed when applying hard-clipping, by instead specifically targeting potential `C>T` and `G>A` transition sites on both the 5′ and 3′ end of the read, respectively. In other terms, this method may be regarded as a conservative compromise between *post-mortem* damage rescaling methods such as mapDamage2, PMDtools, or ATLAS [43–45] and hard-clipping methods such as the trimBam module of the bamUtil software [41]. Required inputs for `pmd-mask` are as follows:

- `--bam`: An input `.bam` file (SAM, BAM, and CRAM formats are accepted). `pmd-mask` can either read from a file (using `-b|--bam`) or from the standard input, through shell piping.
- `--misincorporation`: A `mapDamage2` output `misincorporation.txt` file. This file provides strand-specific PMD frequency estimates, which are used to compute the threshold at which masking should be performed. Evidently, this file must have been generated from the input BAM file to provide a sound estimate.
- `--reference`: A reference genome, in the form of a `.fasta` file. This genome must of course be identical to the one used to align the input BAM file.

Additional instructions regarding the installation and usage of `pmd-mask`, as well as its source code is made publicly available at https://github.com/MaelLefeuvre/pmd-mask, under GPL-v3.0 licencing.

2.2 Pseudo-code describing the main algorithm of pmd-mask

Algorithm 1 Pseudo-code describing the main algorithm of pmd-mask.

```
Ensure: : args.threshold ∈ [0.00, 1.00[
bam ← bam_reader(args.bam_path);
refseq ← fasta_reader(args.reference_path)
misincorporation ← misincorporation_reader(args.misincorporation_path)

if args.threshold is not null then
    threshold ← args.threshold
else
    threshold ← 0.01

function MASK(read, position)
    read.nucleotide[position] ←' N';
    read.quality[position] ← 0

output_bam ← bam.copy_header()
for read ∈ bam do
    ▷ Extract read length, coordinate and strand information ◁
    n ← read.length
    chr ← read.chromosome
    pos ← read.position
    strand ← read.strand
    ▷ Mask 5' Cytosines ◁
    for mask5' : (i = 0; i < n; i + +) do
        if misincorporation['C > T'][chr][strand][i] ≤ args.threshold then
            break mask5'
        else if reference.get(chr, pos + i) ==' C' then
            MASK(read, i)
    ▷ Mask 3' Guanines ◁
    for mask3' : (i = n; i > 0; i − −) do
        if misincorporation['G > A'][chr][strand][i] ≤ args.threshold then
            break mask3'
        else if reference.get(chr, pos + i) ==' G' then
            MASK(read, i)

    ▷ Store masked read ◁
    output_bam+ = read
  return (output_bam)
```

3 List of key parameters

Most of BADGER's parameters and flags are provided using configuration files in the YAML format, which are then fed by BADGER to the various tools and methods comprising this pipeline. All configuration files used to generate the simulations of this benchmark are available within the Zenodo archive associated with this manuscript, and can be used to fully replicate the results presented here. For the sake of convenience, however, a subset of these parameters is described here. This subset corresponds to the set of key parameters that were traversed at least once by the various directed acyclic graphs (DAG) of computational tasks, constructed by BADGER during this benchmark.

- All parameters that remained constant during this benchmark are displayed here in black, while those that are part of the benchmark parameter space are displayed in red. These variable parameters are additionally marked as a list – with each modality separated by a comma – and enclosed in curly braces.
- The parameters corresponding to files can either be consulted directly in BADGER's source-code repository (see Section Data availability), or are automatically downloaded by BADGER, prior to simulations.

```
ped-sim:
  replicates:             10
  filter-indels:          False
  filter-maf:             False
  data:
    codes:                {"outbred/pedigree_codes.txt", "inbred/pedigree_codes.txt"}
    definition:           {"outbred/pedigree.def", "inbred/pedigree.def"}
    map:                  "recombination_maps/refined_mf.simmap"
    interference:         "interference_maps/nu_p_campbell.tsv"
  params:
    error-rate:           0
    missingness:          0
    retain-extra:         0
    pop:                  {"CEU", "PUR", "CLM", "MXL", "PEL", "ASW"}
gargammel:
  coverage:               {0.02, 0.04, 0.06, 0.08, 0.10, 0.20, 0.50, 1.00}
  comp-endo:              {1.00, 0.99, 0.98, 0.97, 0.96, 0.95, 0.90}
  comp-cont:              {0.00, 0.01, 0.02, 0.03, 0.04, 0.05, 0.10}
  comp-bact:              0.00
  pmd-model:              "misincorporation"
  misincorporation:
    file:                 "misincorporations/Chan_meso/misincorporation.txt"
    protocol:             "double"
```

```
  sizefreq:             "sizefreqs/Chan_Meso-sizefreq.txt"
  qshift:               0
  params:
    contam-pop:         "AFR"
preprocess:
  trimming:
    min-overlap:        1
    min-length:         17
    min-quality:        20
    qualitymax:         41
  bwa:
    aligner:            "aln"
    collapsed-only:     False
    bwa-aln:
      seed-length:      1024
      max-open-gap:     2
      max-seed-diff:    2
      max-miss-prob:    0.01
  filter:
    min-MQ:             20
    min-length:         30
  dedup:
    method:             picard
  pmd-rescaling:
    rescaler:           {" ", "mapdamage"}
    apply-masking:      {" ", "pmd-mask", "trimbam"}
    map-damage:
      rescale:          {"False", "True"}
    pmd-mask:
      threshold:        0.01
    trimbam:
      trim-length:      10
      ignore-strand:    False
      soft-clip:        False
variant-calling:
  caller:               "pileupCaller"
  maf:                  0.00
  maf-superpop:         "EUR"
  pileup:
    disable-BAQ:        True
    min-BQ:             20
    min-MQ:             20
  pileupCaller:
    skip-transitions:   False
    mode:               "randomHaploid"
    min-depth:          1
kinship:
  targets:              "aadr_v52.2_1240K_public.snp"
  exclude-samples:      []
  READ:
    window-size:        1000000
    norm-method:        "median"
  READv2:
    window-est:         False
    window-size:        5000000
    norm-method:        "median"
    2pow:               False
  GRUPS:
    pedigree:           "grups-rs/pedigrees/siblings-pedigree.ped"
    pedigree-pop:       "EUR"
    contam-pop:         "EUR"
    contam-rate:        0.0
    min-depth:          1
    mode:               "fst"
    reps:               1000
    maf:                0.0
    min-qual:           30
    seq-error-rate:     0.0
  TKGWV2:
    downsample:         False
    target-frequencies: "TKGWV2/1240K/1000GP3_EUR_1240K.frq"
    min-BQ:             30
    min-MQ:             20
    min-overlap:        1
```

```
KIN:
  interval:           10000000
  p0-threshold:       10
  contam-parameter:   0
correctKin:
  deplete-indivs:     False
  reference-pop:      "EUR"
```

4 Key Resources Table

Reagent or Resource	Source	Identifier
**Deposited data**		
1000g-phase3-v20130502	IGSR [32]	https://ftp.1000genomes.ebi.ac.uk/vol1/ftp/release/20130502/
HapMapII	IHMP [65]	http://ftp.ncbi.nlm.nih.gov/hapmap/recombination/2011-01_phaseII_B37/
GRCh37-release113	Church et al. [57]	http://ftp.ensembl.org/pub/grch37/release-113/fasta/homo_sapiens/dna/
Cross-over interference model	Campbell et al. [54]	https://raw.githubusercontent.com/williamslab/ped-sim/refs/heads/master/interfere/nu_p_campbell.tsv
Sex-specific genetic maps	Bhérer et al. [53]	https://github.com/cbherer/Bherer_etal_SexualDimorphismRecombination/
AADR dataset v52.2	Mallick et al. [60]	https://reichdata.hms.harvard.edu/pub/datasets/amh_repo/curated_releases/V52/V52.2/SHARE/public.dir/
TKGWV2 support files	Fernandes et al. [19]	https://github.com/danimfernandes/tkgwv2
**Softwares and algorithms**		
AdapterRemoval-v2.3.3	Schubert et al. [56]	RRID:SCR_011834
ANGSD-v0.939	Korneliussen et al. [62]	RRID:SCR_021865
BADGER-v0.5.2	This study	https://github.com/MaelLefeuvre/badger/tree/v0.5.1
bamUtil-v1.0.15	Jun et al. [41]	RRID:SCR_027781
bcftools-1.15	Li [52]	RRID:SCR_005227
conda-23.1.0	Anaconda Inc.	RRID:SCR_018317
correctKin	Nyerki et al. [22]	RRID:SCR_026952
gargammel-1.1.4	Renaud et al. [37]	RRID:SCR_026953
grups-rs-0.3.2	Lefeuvre et al. [24]	RRID:SCR_026954
kin-3.1.3	Popli et al. [21]	RRID:SCR_026955
mapDamage-v2.2.1	Jónsson et al. [43]	RRID:SCR_001240
pcangsd-0.99	Meisner and Albrechtsen [64]	RRID:SCR_026956
ped-sim-v1.4	Caballero et al. [36]	RRID:SCR_026957
picard-v2.27.4	Broad Institute [59]	RRID:SCR_006525

plink-v1.9	Chang et al. [63]	RRID:SCR_001757
pmd-mask-v0.3.2	This study	https://github.com/MaelLefeuvre/pmd-mask/tree/v0.3.2
READ-v1.0	Kuhn et al. [16]	RRID:SCR_026958
READv2-v2.00	Alaçamlı et al. [25]	RRID:SCR_026959
samtools-v1.15	Li [52]	RRID:SCR_002105
sequenceTools-v1.5.2	Schiffels [61]	https://github.com/stschiff/sequenceTools/tree/v1.5.2
snakemake-7.20.0	Mölder et al. [35]	RRID:SCR_003475
TKGWV2	Fernandes et al. [19]	RRID:SCR_026960
python-3.11.0	Python Software Foundation	RRID:SCR_008394
R-v4.1.2	R Development Core Team	RRID:SCR_001905
